# Gonadal Transcriptome Alterations in Response to Dietary Energy Intake: Sensing the Reproductive Environment

**DOI:** 10.1371/journal.pone.0004146

**Published:** 2009-01-07

**Authors:** Bronwen Martin, Michele Pearson, Randall Brenneman, Erin Golden, William Wood, Vinayakumar Prabhu, Kevin G. Becker, Mark P. Mattson, Stuart Maudsley

**Affiliations:** 1 Laboratory of Neurosciences, National Institute on Aging, Baltimore, Maryland, United States of America; 2 Gene Expression and Genomics Unit, National Institute on Aging, Baltimore, Maryland, United States of America; 3 Receptor Pharmacology Unit, National Institute on Aging, Baltimore, Maryland, United States of America; Cincinnati Children's Research Foundation, United States of America

## Abstract

Reproductive capacity and nutritional input are tightly linked and animals' specific responses to alterations in their physical environment and food availability are crucial to ensuring sustainability of that species. We have assessed how alterations in dietary energy intake (both reductions and excess), as well as in food availability, via intermittent fasting (IF), affect the gonadal transcriptome of both male and female rats. Starting at four months of age, male and female rats were subjected to a 20% or 40% caloric restriction (CR) dietary regime, every other day feeding (IF) or a high fat-high glucose (HFG) diet for six months. The transcriptional activity of the gonadal response to these variations in dietary energy intake was assessed at the individual gene level as well as at the parametric functional level. At the individual gene level, the females showed a higher degree of coherency in gonadal gene alterations to CR than the males. The gonadal transcriptional and hormonal response to IF was also significantly different between the male and female rats. The number of genes significantly regulated by IF in male animals was almost 5 times greater than in the females. These IF males also showed the highest testosterone to estrogen ratio in their plasma. Our data show that at the level of gonadal gene responses, the male rats on the IF regime adapt to their environment in a manner that is expected to increase the probability of eventual fertilization of females that the males predict are likely to be sub-fertile due to their perception of a food deficient environment.

## Introduction

The availability of energy in the form of food is a critical factor in the maintenance of the reproductive capacity of mammals. Low, moderate and high levels of dietary energy intake can affect reproductive function in different ways. Maintaining reproductive capacity is an energy-consuming process which is tightly regulated on a gonadal transcriptional level. It is presently unclear how dietary energy intake affects gonadal gene regulation. We have shown previously that low, moderate and high energy diets affect male and female rats differently on biochemical, endocrine, behavioral and genetic levels [1], [2]. At a fundamental level, the maintenance of reproductive capacity is the most crucial function that animals need to maintain and, therefore, the capacity to identify and secure energy in the form of food is essential to maintain reproductive status.

We have previously shown that alterations in caloric intake in rats had significant effects upon the female reproductive axis [1]. This altered reproductive state was also connected to an increase in cognitive capacity and activity levels, changes expected to improve the chances of females securing food in their environment. Their apparent masculinization in response to extreme caloric restriction probably occurred to aid their competition (with other males or females) for any available food sources. In the females there seemed to be a specific capacity inherent to their hippocampal transcriptional activity to sense their nutritional input. The corresponding males showed no calorie-restriction specific hippocampal transcriptome sensitivity [2]. In this context, at the level of the hippocampus, it appears that males and females seem to engineer a competition between each other for the remaining available food, *i.e.* the females ‘masculinize’ to aid their competition with any resident males. However for animals that generate offspring via sexual reproduction, a cooperativity of activity and response to environmental food availability would be critical. It would be biologically wasteful for either gender to maintain a contemporaneous high reproductive capacity while their opposite gender does not. Whether this linkage is retained in animals that have not been in contact with the opposite gender or have mated is an interesting postulate. It has been shown to some extent that reciprocal ‘anticipation’ of each other's gender's fecundity occurs in natural settings. For example, in free-living spotted hyenas (*Crocuta crocuta*) their reproductive status (number of successful births) is controlled to some extent by seasonality but much more strongly by variation in their environmental energy availability [3], as this would determine how many fertile females were available to mate.

The aim of this study was to identify gender differences in the gonadal transcriptional responses to alterations in the available food energy. To do this, we used multiple dietary paradigms that include calorie restriction (20% CR and 40% CR), every other day feeding (intermittent fasting: IF) and an excessive energy intake diet (high fat/high glucose: HFG). We have elucidated the gonadal transcriptome responses to these different levels of energy intake on a single gene level and on a parametric functional group level. Our findings suggest molecular mechanisms by which males and females differentially modify their reproductive capacity based on the amount and frequency of food availability.

## Results

### Gonadal transcriptional and physical responses to caloric restriction, dietary excess and intermittent fasting

**Figure 1:**
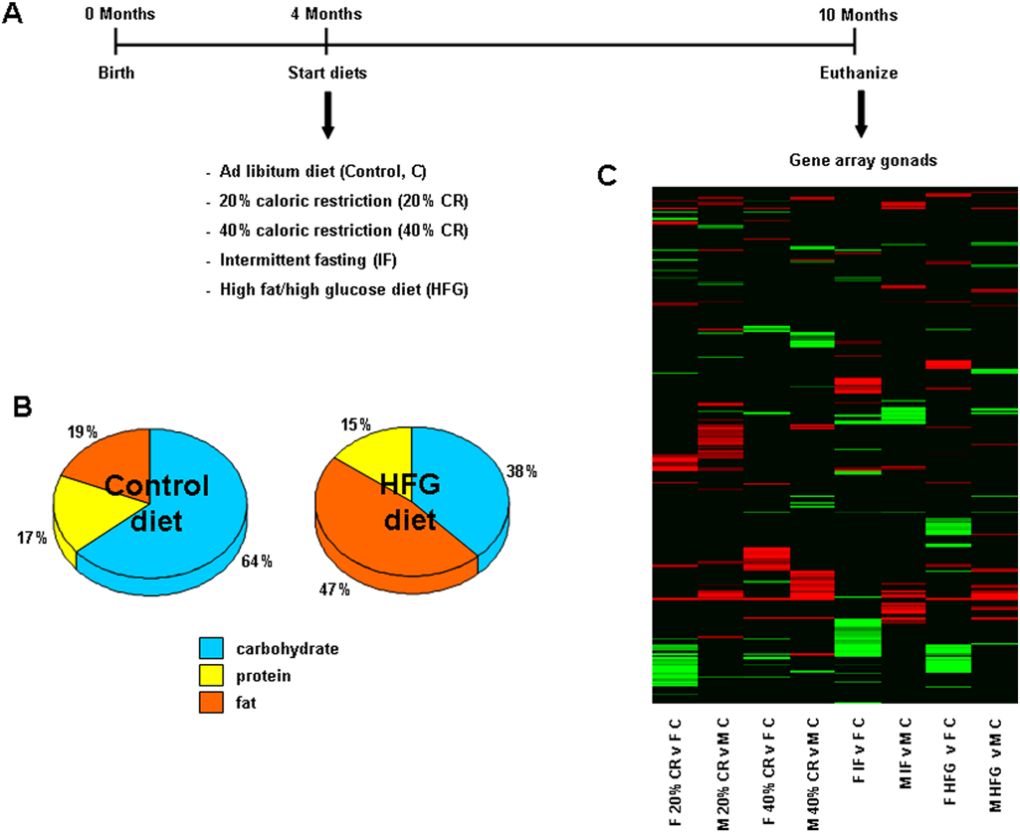
Experimental design, diet composition and heatmap of significantly altered gonadal genes. (A) The experimental timeline for this study. Four-month-old male and female rats were placed on one of 5 dietary regimes (control (ad libitum), 20% caloric restriction (CR), 40% CR, intermittent fasting (IF), or high fat/high glucose (HFG)). At 10 months of age, rats were euthanized and gonadal tissues were collected. A 17 K mouse gene array was performed and significant gene and pathway expression changes were quantified. (B) The relative proportions of the major nutritional groups in the control and high-fat/glucose (HFG) diets. (C) Regulatory heatmap of the significantly up-regulated (red) and down-regulated (green) genes in gonads collected from male and female rats on the different dietary regimes, compared to gonads collected from *ad libitum* controls.

Male and female Sprague Dawley rats were divided into five diet groups: control (*ad libitum*), 20% CR, 40% CR, IF and HFG at four months of age (8–15 animals per group; Figure 1A). The rats in the first four diet groups were fed a diet with a typical composition in which the majority of calories were from complex carbohydrates, whereas the HFG diet contained higher amounts of fat and glucose (Figure 1B). Rats were maintained on the diets until 10 months of age (6 months total). Upon completion of the study, rats were euthanized and the gonads (ovaries and testes) were carefully dissected out and collected for transcriptome analyses. The gonadal gene changes of rats on the different dietary regimes were compared to gonadal genes from the *ad libitum* control rats and significant gene alterations (up- or down-regulation) were reported. The transcriptional heatmap (Figure 1C) represents the variety of significantly up- (red) and down- (green) regulated genes that were altered between the diets in the gonads of the male and female rats.

**Figure 2:**
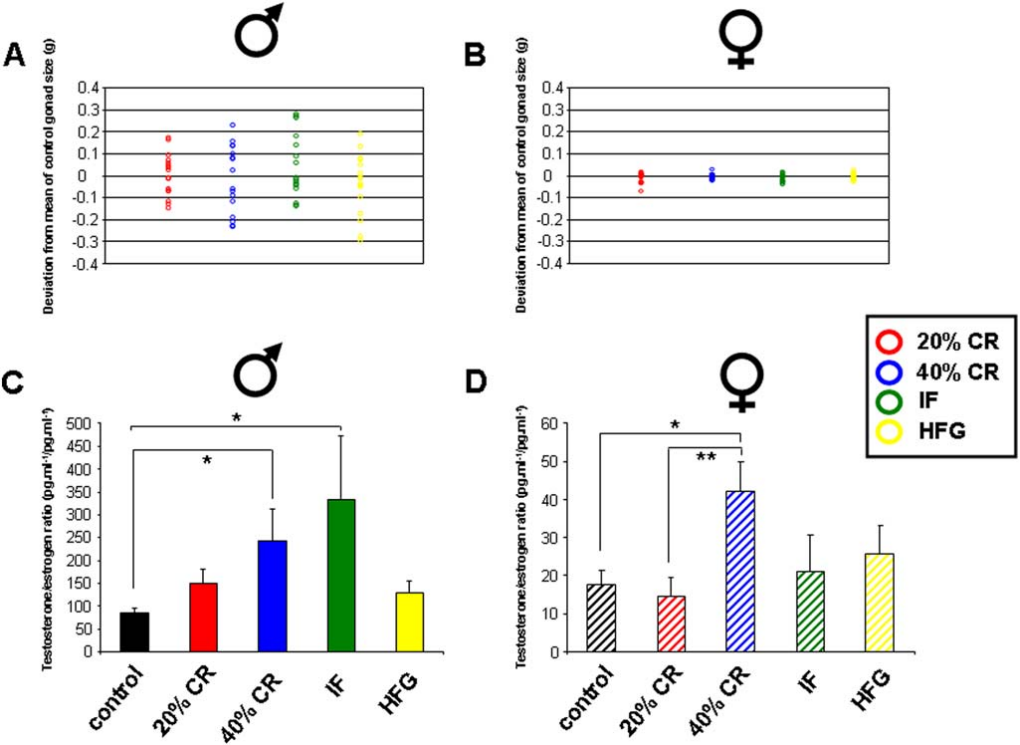
Effects of dietary regimes upon gonadal structure and plasma steroid levels. Panels A and B depict the deviations in grams from the mean mass of the control *ad libitum* animals' gonadal size of the wet masses of the testes (A) or ovaries (B) from the multiple animals (n = 8) on the respective dietary regimes. Panels C and D depict the plasma testosterone/estrogen ratios (pg.ml^−1^/pg.ml^−1^) values for males (C) or females (D) subjected to the respective dietary regimes. Data is represented as mean±S.E. mean, n = 8 and *p<0.05, **p<0.01.

In addition to our investigation into the gonadal transcriptional responses to dietary alterations, we also measured potential alterations in gonadal mass (testes and ovaries) and in the plasma ratios of the two primary sex steroid hormones, testosterone and estrogen. Measurements for the combined wet mass of the left and right gonads of all the animals (male and female) in the multiple study groups showed that, in general, female variation in gonadal size was negligible compared to that of the males (Figures 2A–B). To measure the gonadal size variation compared to control, the mean mass deviation from the male or female control diet mean mass was calculated. To maintain an independence of analysis from the rest of the body, we used non-normalized gonadal mass for this instance. For the males the most extreme dietary regimes (40% CR and HFG), with respect to input calories, tended to induce a reduction in total gonadal mass. The 40% CR and the HFG mean deviations from the control average were −14.8±30 mg and −39±30 mg respectively (both n = 8). The greatest mean increase in mass from control was seen with the IF group (44.3±30 mg, n = 8) followed by a smaller increase in mass compared to control from the 20% CR group (2.75±24 mg, n = 8). The large variation in gonadal size prevented statistical significance with respect to raw wet mass. The female gonadal mass variation in comparison to the males was negligible and was therefore potentially indicative of a specificity of male gonadal responsivity to environmental food availability. Additionally, measurement of the testosterone/estrogen (T/E) ratios (pg.ml^−1^/pg.ml^−1^) showed a similar pattern to the gonadal size alterations in response to the variations in energy intake, *i.e.* the IF group showed the largest increase in mean T/E ratio (Figure 2C). The 40% CR diet induced a modest increase in T/E ratio while the 20% CR and HFG diets induced only minimal increases in T/E ratio. In contrast to the male findings, only the 40% CR diet induced a considerable elevation in the T/E ratio in females, approaching male control levels of T/E ratio (Figure 2D).

### Gender-specific alterations in gonadal gene transcription in response to energy restriction and excess

**Figure 3:**
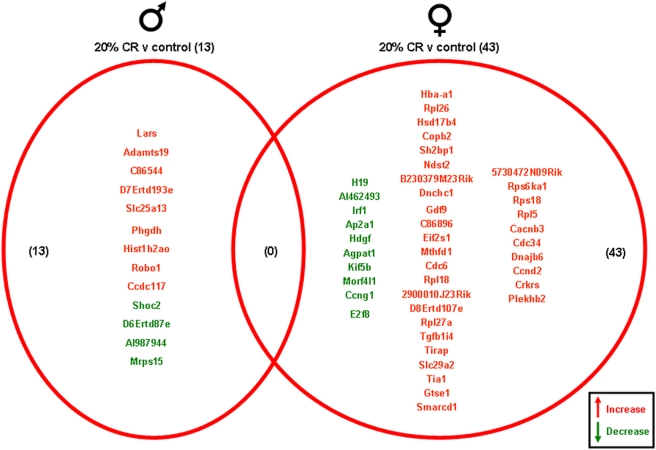
Gene changes in the gonads of male and female rats maintained on the 20% CR diet compared to the gonads of male and female rats maintained on a control (*ad libitum*) diet. Genes that were significantly up-regulated (red) or down-regulated (green) were clustered into a Venn diagram. There were 9 significantly up-regulated and 4 significantly down-regulated genes in the testes collected from 20% CR male rats compared to the genes from testes collected from control (*ad libitum*) male rats. Ovaries collected from 20% CR female rats showed 33 significantly up-regulated (red) and 10 significantly down-regulated (green) genes compared to control (*ad libitum*) female rats. There were no common gene alterations between the 20% CR male and female gonadal tissues. Names of the significantly altered genes can be found in Table S1.

**Figure 4:**
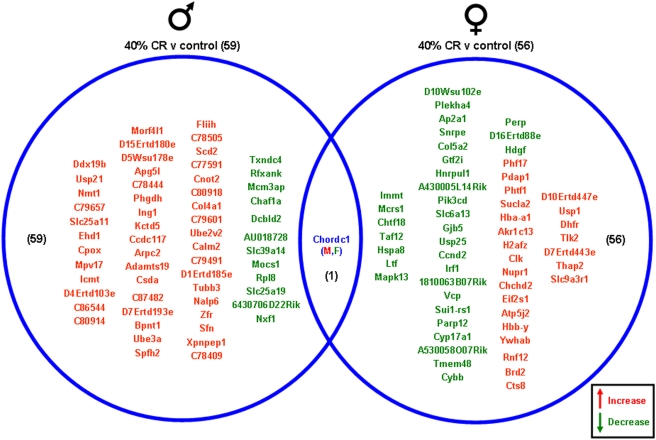
Gene changes in the gonads of male and female rats maintained on a 40% CR diet compared to the gonads of rats maintained on a control (*ad libitum*) diet. Genes that were significantly up-regulated (red) or down-regulated (green) were clustered into a Venn diagram. There were 47 significantly up-regulated and 12 significantly down-regulated genes in the testes collected from 40% CR male rats compared to controls. Ovaries collected from 40% CR female rats showed 24 significantly up-regulated (red) and 32 significantly down-regulated (green) genes compared to the control *ad libitum* females. There was 1 common gene altered in the gonads of both the male and female rats in this dietary group. This gene, chordc1, was up regulated in female gonadal tissue and down regulated in male gonadal tissue. Names of the significantly altered genes can be found in Table S1.

**Figure 5:**
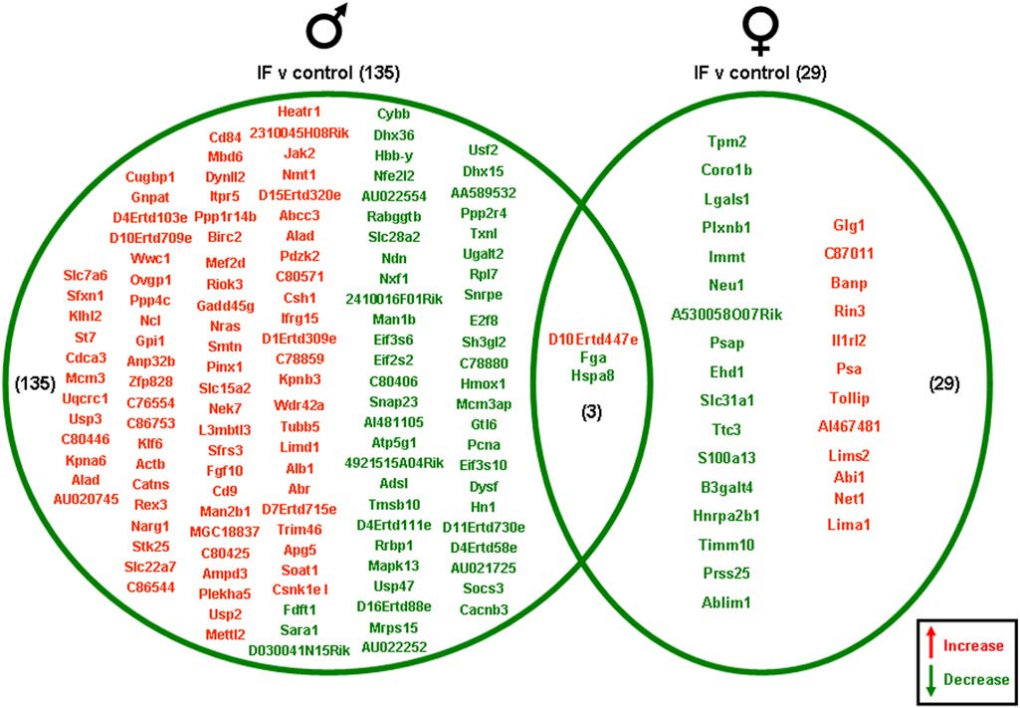
Gene changes in the gonads of male and female rats maintained on an IF diet compared to the gonads of rats maintained on a control (*ad libitum*) diet. Genes that were significantly up-regulated (red) or down-regulated (green) were clustered into a Venn diagram. There were 82 significantly up-regulated and 53 significantly down-regulated genes in the testes collected from IF male rats compared to the genes from testes collected from control (*ad libitum*) male rats. Ovaries collected from IF female rats showed 12 significantly up-regulated (red) and 17 significantly down-regulated (green) genes compared to controls. There were 3 genes that was significantly altered in both the males and females in the IF dietary group compared to the control group. The expressed sequence D10Ertd447e was significantly up-regulated in the gonads of IF males and females, compared to control (*ad libitum*) males and females. The genes Fga and Hspa8 were each down-regulated in IF male and female gonads compared to controls. Names of the significantly altered genes can be found in Table S1.

**Figure 6:**
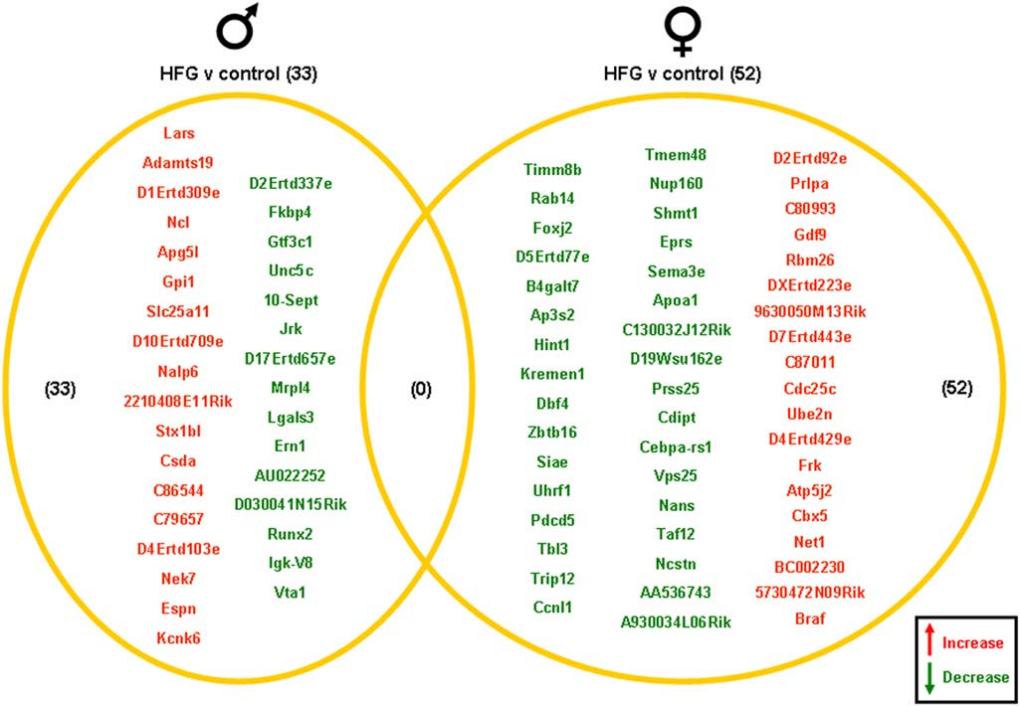
Gene changes in the gonads of male and female rats maintained on the HFG diet compared to the gonads of male and female rats maintained on a control (*ad libitum*) diet. Genes that were significantly up-regulated (red) or down-regulated (green) were clustered into a Venn diagram. There were 18 significantly up-regulated and 15 significantly down-regulated genes in the testes collected from HFG male rats compared to controls. Ovaries collected from HFG female rats showed 19 significantly up-regulated (red) and 33 significantly down-regulated (green) genes compared to controls. There were no common genes between male and female rats in this dietary group. Names of the significantly altered genes can be found in Table S1.

Significantly altered genes in the gonads of the male and female rats on the various dietary regimes compared to genes from the gonads of the *ad libitum* controls are summarized in the Venn diagrams (Figure 3, 4, 5, 6). The 20% CR males had 13 significant gonadal gene alterations, compared to *ad libitum* male controls. Of these significantly altered genes, 9 were significantly up-regulated and 4 were significantly down-regulated. The 20% CR females had 43 significantly altered gonadal genes compared to control *ad libitum* females, approximately 3 times more than the 20% CR males. Of these 43 significantly altered genes, 33 were up-regulated and 10 were down-regulated (Figure 3). The 40% CR males had 59 significantly altered genes compared to male controls; 47 were up-regulated and 12 were down-regulated. The 40% CR females had 56 significantly altered gonadal genes compared to controls, and 24 of these genes were up-regulated and 32 were down-regulated. One gene, Chordc1 (cysteine and histidine-rich domain-containing zinc-binding protein 1), was differentially altered between the gonads of the male and female 40% CR rats. This gene was up-regulated in the female gonadal tissue and down-regulated in the male gonadal tissue (Figure 4). Chordc1 is a calcium and zinc ion binding protein and is thought to interact with heat shock protein 90 [4]. The IF males had 135 significantly altered genes compared to the *ad libitum* male controls. Of these 135 genes, 82 were up-regulated and 53 were down-regulated. The IF females had 29 significantly altered gonadal genes compared to the control females; 12 of these genes were up-regulated and 17 were down-regulated. Additionally, three genes were significantly altered in both the male and female gonadal tissues. The expressed sequence D10Ertd447e was significantly up-regulated in the gonads of both the IF males and females. The genes Fga (fibrinogen, alpha polypeptide) and Hspa8 (heat shock protein 8) were each down-regulated in IF male and female gonadal tissues compared to *ad libitum* controls (Figure 5). Males on the HFG diet had 33 significantly altered genes compared to *ad libitum* male controls. There were 18 up-regulated genes and 15 down-regulated genes. HFG females had 52 significantly altered gonadal genes compared to female controls. Of these genes, 19 were up-regulated and 33 were down-regulated. There were no commonly altered genes in the gonads of males and females on the HFG diet (Figure 6).

### Calorie-restriction specificity of gonadal gene regulation

**Figure 7:**
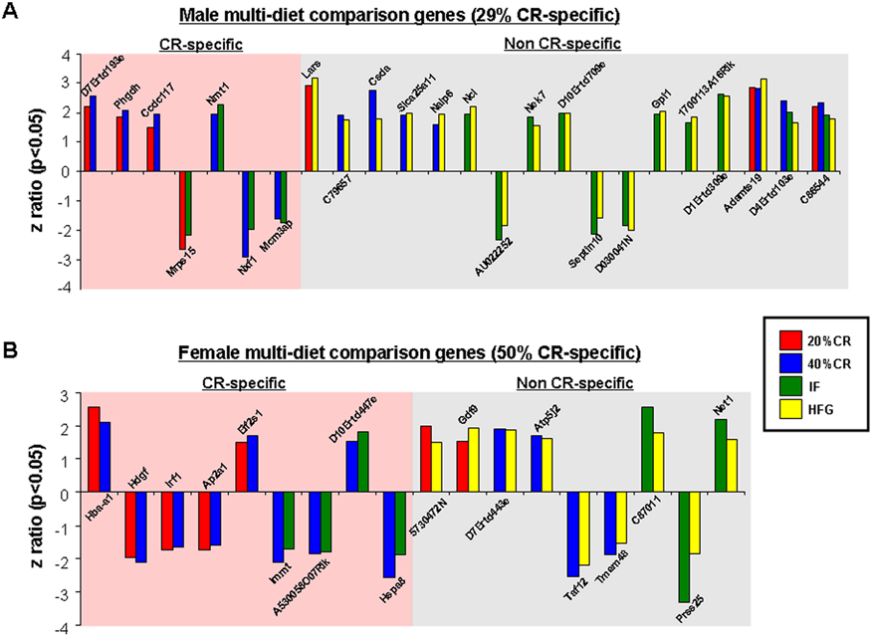
Caloric specificity of male and female multi-diet regulated genes. Significantly altered genes that were common between one or more of the diet paradigms are drawn to visualize caloric specificity. (A) Commonly altered genes in the testes of male rats on the various diets showed 29% caloric restriction (CR) specificity (commonly regulated between the 20% CR, 40%, CR, and/or IF diets only) and 71% non-CR specificity. (B) Commonly altered genes in the ovaries of female rats on the various diets showed 50% caloric restriction (CR) specificity and 50% non-CR specificity. Names of the significantly altered genes can be found in Table S1.

**Figure 8:**
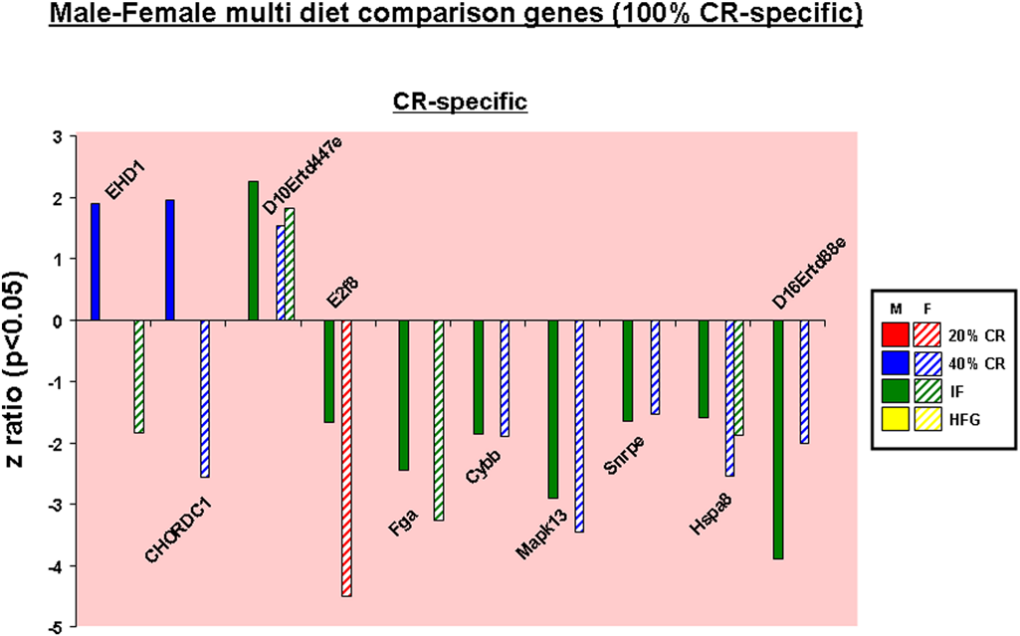
Caloric specificity of male-female multi-diet regulated genes. Significantly altered genes that were common between one or more of the diet paradigms in the ovaries and the testes were drawn to visualize caloric specificity in cross-gender regulated genes. Names of the significantly altered genes can be found in Table S1.

Significant gene alterations in the gonads of both genders were compared across the multiple dietary paradigms using a 4-way Venn-diagram (Figures S1 & S2). Gonadal gene changes were analyzed further to determine commonalities between high and low energy diet groups (Figures 7–8). In a previous study, we found that the hippocampal genetic response to dietary restriction and excess was calorie-dependent in females and calorie-independent in males at both the single gene and functional pathway levels [2]. In the testes, only 29% of the multi-dietary paradigm-regulated genes were CR-specific, *i.e.* were not significantly regulated (up or down) in the male HFG group (Figure 7A). Amongst the multi-diet regulated genes in the testes, there was a preponderance of genes involved in energy regulation (Phgdh, Mrps15), post-translational modification (Nmt1), protein complex regulation and transport (Csda, Nalp6, Septin 10) and most importantly nucleic acid synthesis/regulation (Mcm3ap, Lars, Ncl, Nek7). In contrast to the testes, 50% of the multi-dietary paradigm-regulated genes in the ovaries were CR-specific (Figure 7B). Amongst the significantly regulated genes there appeared to be clusters related to growth factor action (Hdgf, Gdf9), mitochondrial function (Immt, Prss25) and translation (Eif2s1, Taf12).

When multi-diet cross-gender comparisons of co-regulated genes were performed, we found that this specific gene-set was entirely CR-specific (Figure 8). Eighty percent of this specific gene set was co-regulated coherently between males and females, *i.e.* no difference in the direction of gene regulation. Seventy percent of this gene-set was however coherently down-regulated. The EH-domain-containing protein 1 (EHD1) gene was up-regulated in males yet down-regulated in females; this protein has been implicated in the regulation of vesicle transport [5], [6] and cholesterol homeostasis [7]. A similar pattern was noted for the Chordc1 gene [4]. As mentioned previously, Chordc1 encodes for a protein involved in forming heat shock protein complexes that can then interact with ATPases and protein phosphatases to modulate the activity of Nod1 in its role in innate immune responses [8]. Amongst the common down-regulated genes, there was a general predominance of genes normally involved in cell stress/death (Cybb, Mapk13, Hspa8) or transcriptional regulation (E2f8, Snrpe).

### Gonadal functional gene pathways are differentially altered in males and females in response to dietary energy restriction and excess

**Figure 9:**
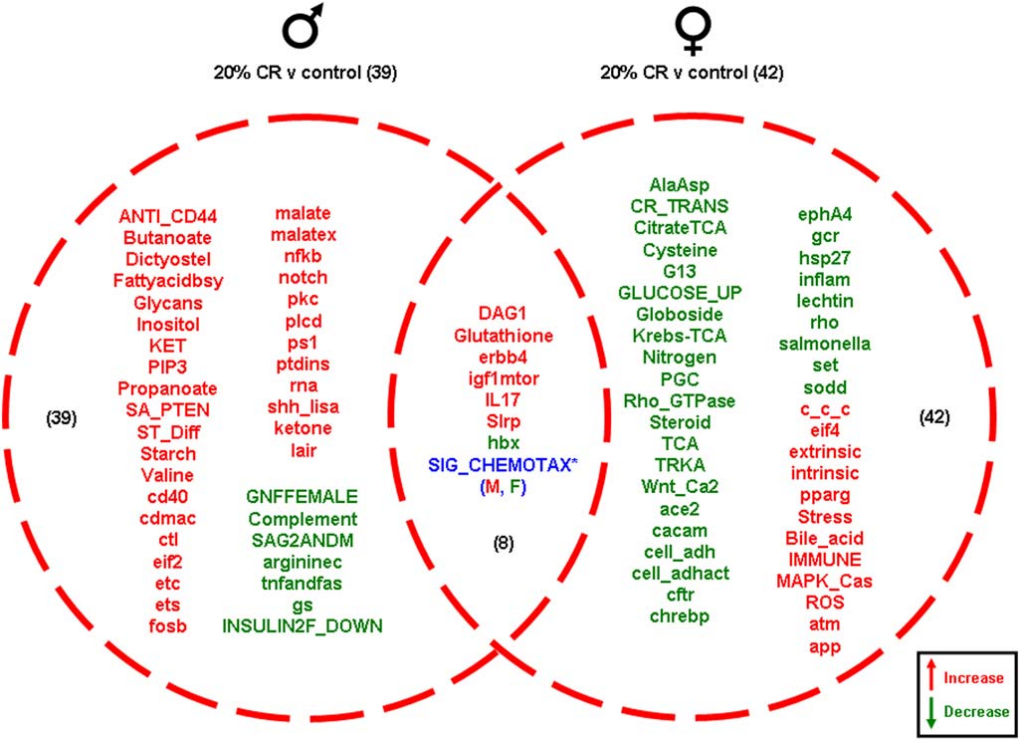
Pathway changes in the gonads of male and female rats maintained on the 20% CR diet compared to the gonads of male and female rats maintained on the control (*ad libitum*) diet. Significantly regulated, functional pathway clusters were generated from the respective male or female gene sets using PAGE gene set analysis. Pathways that were significantly up-regulated (red) or down-regulated (green) were clustered into a Venn diagram. Pathways in blue were common to both males and females but were differentially regulated, *e.g.* the SIG_CHEMOTAX pathway, was differentially altered in the 20% CR male (M) and female (F) gonads. This pathway was up-regulated in the males and down regulated in the females in comparison to *ad libitum* controls. Names of the significantly altered pathways can be found in Table S2.

**Figure 10:**
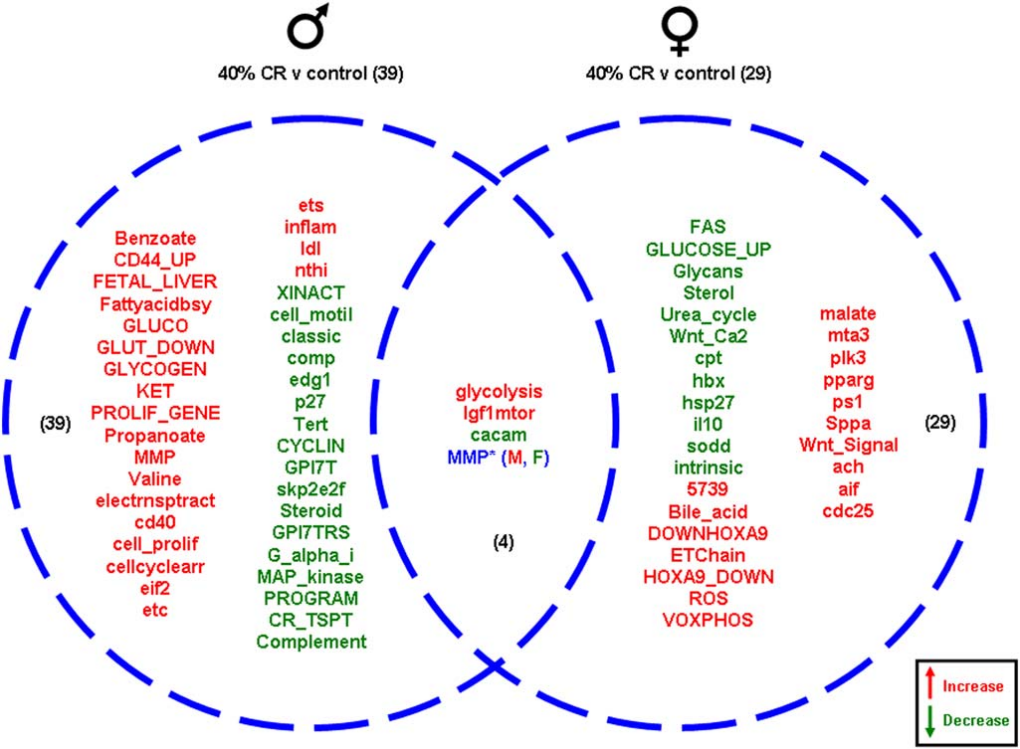
Pathway changes in the gonads of male and female rats maintained on the 40% CR diet compared to the gonads of male and female rats maintained on the control (*ad libitum*) diet. Significantly regulated, functional pathway clusters were generated from the respective male or female gene sets using PAGE gene set analysis. Pathways that were significantly up-regulated (red) or down-regulated (green) were clustered into a Venn diagram. Pathways in blue were common to both males (M) and females (F) but were differentially regulated. Names of the significantly altered pathways can be found in Table S2.

**Figure 11:**
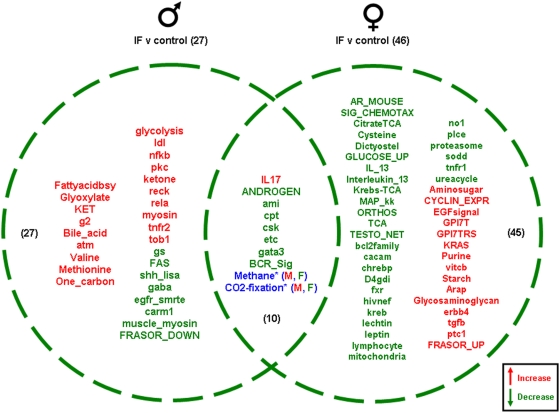
Pathway changes in the gonads of male and female rats maintained on the IF diet compared to the gonads of male and female rats maintained on the control (*ad libitum*) diet. Significantly regulated, functional pathway clusters were generated from the respective male or female gene sets using PAGE gene set analysis. Pathways that were significantly up-regulated (red) or down-regulated (green) were clustered into a Venn diagram. Pathways in blue were common to both males (M) and females (F) but were differentially regulated. Names of the significantly altered pathways can be found in Table S2.

**Figure 12:**
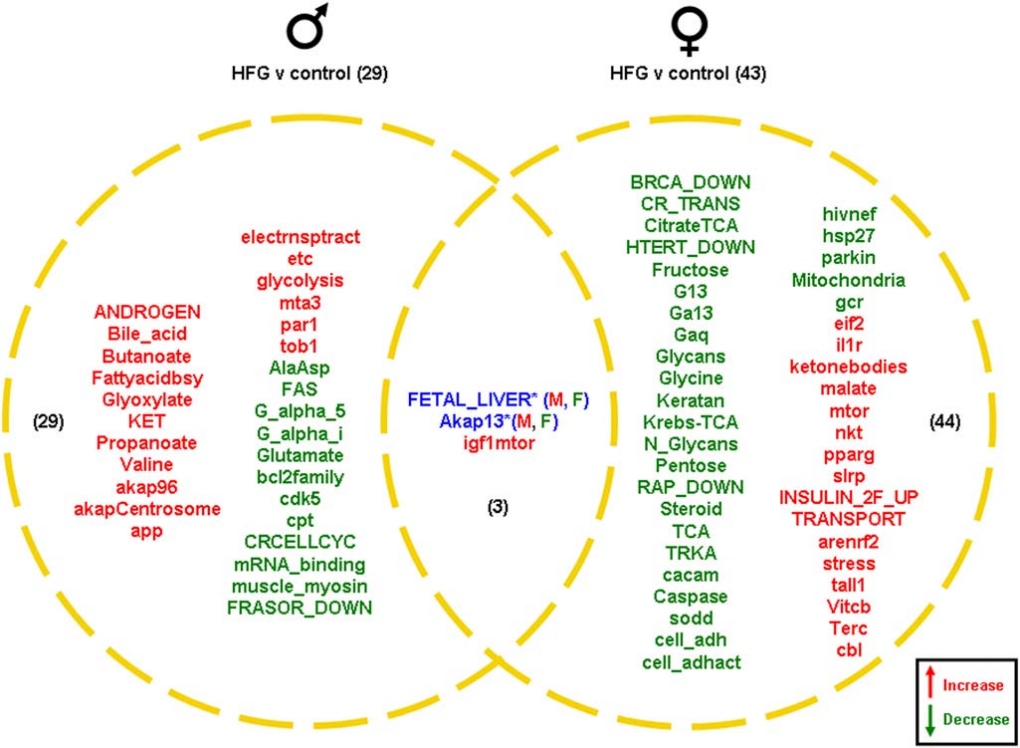
Pathway changes in the gonads of male and female rats maintained on the HFG diet compared to the gonads of male and female rats maintained on a control (*ad libitum*) diet. Significantly regulated, functional pathway clusters were generated from the respective male or female gene sets using PAGE gene set analysis. Pathways that were significantly up-regulated (red) or down-regulated (green) were clustered into a Venn diagram. Pathways in blue were common to both males (M) and females (F) but were differentially regulated. Names of the significantly altered pathways can be found in Table S2.

Significantly altered gonadal genes were grouped into functional pathway categories, established by the Broad Research Institute (Massachusetts Institute of Technology: http://www.broad.mit.edu/gsea/msigdb), to form 522 functional gene pathways. The up- or down-regulation of these functional gene pathways in the ovaries and testes of the rats on the various dietary regimes (compared to *ad libitum* fed controls) was analyzed and is summarized in the Venn diagrams in Figures 9, 10, 11, 12 (ribbon graphs showing the cumulative pathway z-scores are depicted in Figures S3, S4, S5, S6). The 20% CR males had 39 significantly altered pathways compared to control *ad libitum* males; 32 pathways were up-regulated and only 7 were down-regulated. Among the up-regulated pathways unique to the 20% CR, there was a dominance of signaling routes involved in energy metabolism (Butanoate, FA biosynthesis, KET, malate, malatex, ketone), immune function/cytokine activity (ANTI_CD44, nfkb, ctl, notch, lair) and development/differentiation (ST_Diff, cd40, notch, pkc, shh_lisa). Interestingly, the 20% CR females showed an opposite response to the 20% CR males, as the majority of their significantly altered pathways were down-regulated compared to the *ad libitum* control females. Of the 42 significantly altered pathways in the 20% CR ovaries, only 11 were up-regulated and 31 were down-regulated. The down-regulated pathways in the 20% CR females were primarily concerned with stress response (set, sodd, pparg, hsp27, inflamm), steroidal regulation (steroid, gcr, PGC), energy regulation (citrate TCA, GLUCOSE_UP, Krebs-TCA, TCA), development (TRKA, Wnt_Ca2, cftr, ephA4, chrebp) and cellular structure/adhesion (Rho_GTPase, cell_adh, cell_adhact, rho). Additionally, there were 8 pathways that were common between the males and females. There were 6 pathways that were significantly up-regulated in both the 20% CR ovaries and testes compared to the controls, and 1 pathway that was significantly down-regulated in the ovaries and testes. Another pathway, Sig_Chemotax, was significantly up-regulated in the 20% CR testes and significantly down-regulated in the 20% CR ovaries compared to the controls (Figure 9).

The 40% CR males showed 39 significantly altered genetic pathways compared to male controls, and 22 of these pathways were up-regulated and 17 pathways were down-regulated (Figure 10). Among the up-regulated pathways unique to the 40% CR males, certain functional activities were heavily represented, *i.e.* immune/inflammatory function (CD44_UP, cd40, inflam, nthi), energy regulation (FA biosynth, GLUCO, GLUT_DOWN, GLYCOGEN, KET, electrontransport, ets) and cell development (PROLIF_GENE, cell_prolif, cellcyclearrest, eif2). The unique down-regulated pathways observed in 40% CR males included general groups such as cell cycle control (p27, Tert, CYCLIN, skp2e2f), immune/inflammatory function (classic, complement, comp) cell structure (cell_motil, edg1) and GPCR signaling (GPI7T, GPI7TRS, G_alpha_i). The 40% CR females had 29 significantly altered pathways compared to female controls. Of these, 17 pathways were up-regulated and 12 pathways were down-regulated. The unique down-regulated pathways in the 40% CR females included pathways involved in development (Wnt_Ca2, hbx), cytokine activity (FAS, IL10) and stress (hsp27, sod), while the unique up-regulated pathways play a role in energy metabolism (ETchain, VOXPHOS, malate), receptor activity (mta3, ach) and protease activity (ps1, Sppa). There were 4 functional gene pathways common between the 40% CR ovaries and testes. Two of these pathways were up-regulated in both the male and female gonadal tissues and one pathway was down-regulated in the ovaries and testes. The fourth pathway, MMP, was up-regulated in the 40% CR testes and down-regulated in the 40% CR ovaries compared to the controls (Figure 10).

The IF males had 27 significantly altered functional pathways compared to the *ad libitum* male controls; 19 pathways were down-regulated and 8 pathways were up-regulated. The pathways unique to the IF males' genetic response showed a complex response with respect to cytokine activity and immune function. Hence there was an up-regulation of pathways such as nfkb, rela, tnfr2 and tob1 while there was a down-regulation of FAS. Overall these alterations may serve to facilitate the potential survival of gonadal tissue in the males. Additionally, there was a concerted up-regulation of energy-regulatory pathways.

The IF females showed 46 significant unique pathway alterations compared to control females (Figure 11). Unlike the IF males, the majority of the significantly altered pathways were down-regulated in the IF female gonadal tissue. Of the 46 significantly altered genetic pathways, 31 were down-regulated and 15 were up-regulated. Among the pathways up-regulated, the following primary functions were strongly represented: energy regulation/metabolism (Aminosugar, Purine, vitcb, Starch); cell cycle control (CYCLIN_EPXR, ptc1); receptor signaling (EGFsignal, erbb4, GPI7T, GPI7TRS, KRAS); estrogen receptor functioning (FRASOR_UP). The down-regulated pathways play a role in stress responses (no1, sod, mitochondria, bcl2family, tnfr1), steroid activity (AR_MOUSE, TESTO_NET, fxr), energy metabolism (citrateTCA, Krebs-TCA, TCA, ureacycle), cytokine activity (IL_13, interleukin_13, hivnef, tnfr1), chemotaxis/cAMP function (CHEMOTAXIS, dictyostelium, chrebp, cacam, plce, D4gdi) and satiety (leptin). In addition, there were 10 common pathways between the male and female gonadal tissues. One pathway was significantly up-regulated in both the ovaries and the testes and 7 pathways were significantly down-regulated.

Males on the HFG diet had 29 significantly altered functional pathways compared to the controls (Figure 12). Of these 29 pathways, 17 were up-regulated and 12 were down-regulated. The HFG male unique up-regulated pathways showed a strong presence of certain physiological functions, *i.e.* energy regulation/metabolism (Bile_acid, Butanoate, FA biosynth, Glyoxylate, KET, Propanoate, electronstranport, etc, glycolysis), immune regulation (par1, tob1), cell signaling (akap96, akapCentrosome) and steroid receptor activity (ANDROGEN, mta3). The unique down-regulated pathways demonstrated a strong signaling component (G_alpha_5, G_alpha_i, glutamate), cell cycle control (cdk5, CRcellcylce) and steroid receptor signaling (FRASOR_DOWN). In stark contrast, the HFG females showed a reverse in the directionality of pathway activation, *i.e.* many more pathways were down-regulated uniquely compared to the male HFG animals (Figure 12). The HFG females showed 43 significant pathway alterations; 16 pathways were up-regulated and 27 pathways were down-regulated. Pathways down-regulated in the HFG females alone possessed a strong steroidal (BRCA_DOWN, Steroid) phenotype as well as a considerable number of pathways linked to transcriptional regulation (TRKA, cacam, HTERT_DOWN), cell architecture (cell_adh, cell_adhact), signaling (G13, Galpha13, Galphaq, RAP_DOWN), energy regulation (Fructose, Krebs-TCA, Pentose,) and stress responsiveness (caspase, sod, hivnef, parkin, mitochondria, gcr). Interestingly, the up-regulated female HFG pathways also demonstrated a strong predominance of pathways linked to stress responsiveness (pparg, arenrf2, slrp). Therefore it is possible that even within a single tissue there may be bi-directional regulation of certain pathways in different regions of the organ. Other significantly up-regulated pathways present in the HFG females and not in the males, included those involved in cell proliferation/nutrient sensation (mtor, tall1), immune activity (nkt, il1R), energy regulation (ketonebodies, malate, Vitcb) and subcellular trafficking (TRANSPORT, cbl). There were 3 pathways common between the male and female gonadal tissue. One pathway was significantly up-regulated in both the HFG ovaries and HFG testes and 2 pathways were differentially regulated. These 2 pathways, Fetal_Liver and Akap13, were up-regulated in the testes and down-regulated in the ovaries (Figure 12).

### Caloric specificity of gonadal functional gene pathways

**Figure 13:**
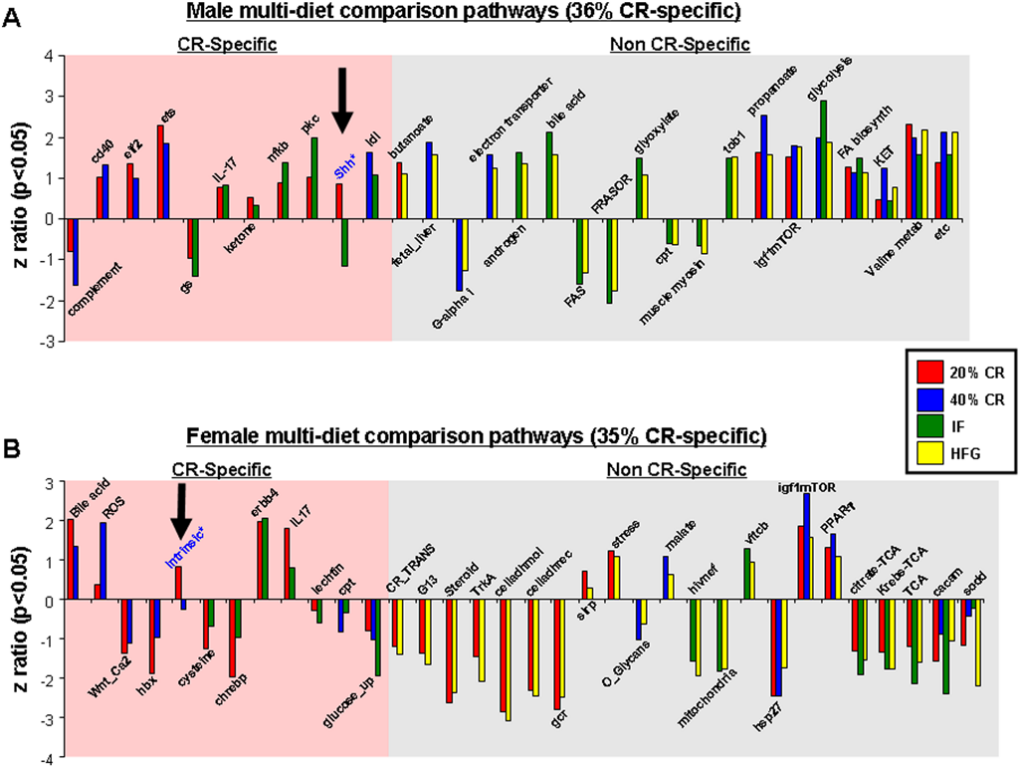
Caloric specificity of male and female multi-diet regulated pathways. Significantly altered functional pathways that were common between diet paradigms were drawn to visualize caloric specificity. (A) Commonly altered functional pathways in the testes of male rats on the various diets showed 36% caloric restriction (CR) specificity (commonly regulated between the 20% CR, 40%, CR, and/or IF diets only) and 64% non-CR specificity. (B) Similarly, the commonly altered functional pathways in the ovaries showed 35% CR-specificity and 65% non-CR specificity. Names of the significantly altered pathways can be found in Table S2.

**Figure 14:**
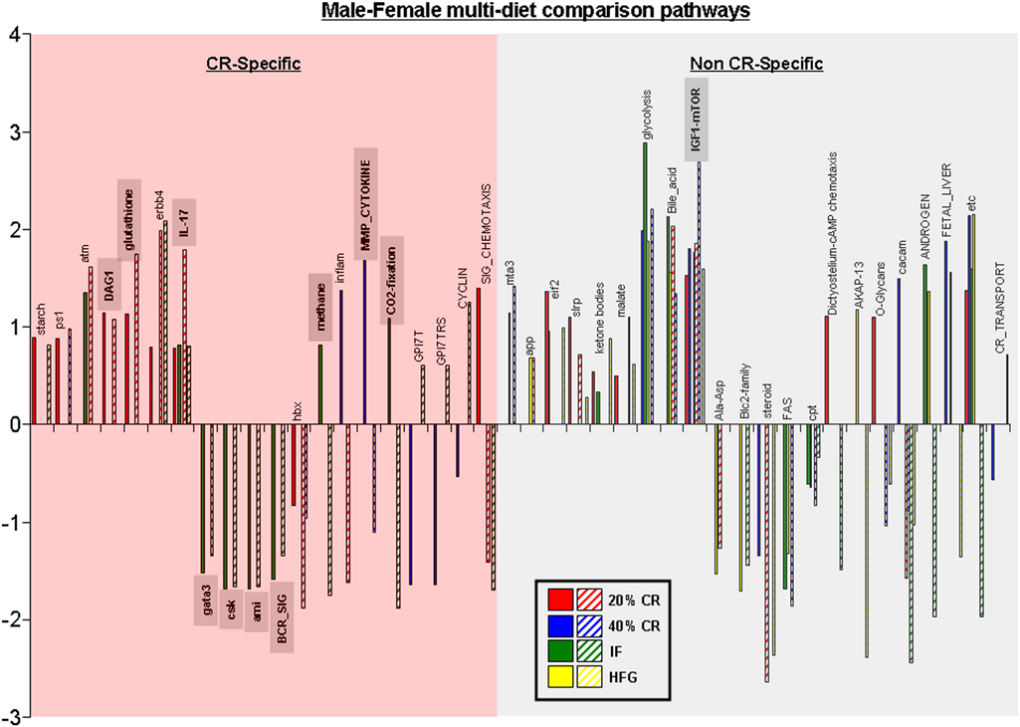
Caloric specificity of male-female common multi-diet regulated pathways. Significantly altered functional pathways that were common between diet paradigms and both genders were drawn to visualize caloric specificity. Names of the significantly altered pathways can be found in Table S2.

Significant functional gene pathway changes in the ovaries and testes were compared across the multiple diet paradigms using a 4-way Venn-diagram (Figures S7 and S8). A representation of the cross-diet pathway regulation combinations is depicted in Figure 13. Interestingly, the percentage of CR-specific multi-diet conserved pathways was almost identical between the males (36%) and the females (35%). In contrast to this similarity in CR-specificity in the multi-diet controlled pathways, the directionality of regulation was considerably different between the two genders, *i.e.* only 23% of male multi-diet pathways were down-regulated while 68% of female multi-diet pathways were down-regulated. Within the genders however, the consistency of directional pathway regulation was almost complete, *i.e.* only one multi-diet regulated pathway was non-uniformly regulated in males (Shh) or females (intrinsic). With respect to the male multi-diet pathways, there was a clear up-regulation of energy/metabolism regulating pathways (ets, ketone, butanoate, electron transport, bile acid, glyoxylate, propanoatem, glycolysis, FA biosynth, KET, valine metab, etc). In contrast, the directionality of regulation of female multi-diet pathways linked to energy/metabolism appeared to be more complex, *e.g.* there was an up-regulation of some pathways (Bile acid, malate, vitcb) while a consistent down-regulation of other energy/metabolism pathways (cysteine, glucose_up, mitochondria, citrate-TCA, Krebs-TCA, TCA). Considering the divergence of gonadal activity between male (consistently active spermatogenesis) and female (cyclicity, controlled conversion between discrete reproductive states) mammals, this is perhaps not a surprising result. When the retention of significantly regulated pathways in common between the transcriptional gonadal responses was analyzed, there were several interesting findings (Figure 14). Representation of the significantly regulated pathways common to more than one dietary regimen between genders, indicates that there was a large number of physiological outcomes that were common between the two genders (42). Almost 48% of these pathways were CR-specific and of these 40% demonstrated a diversity in the direction of regulation between the two genders. This specific subset included pathways regulating energy metabolism (methane, CO_2_ fixation) and receptor signaling (GPI7T, GPI7TRS, SIG_CHEMOTAXIS). Among the non CR-specific cross gender, multi-diet regulated pathways almost 60% of the pathways demonstrated divergence of regulatory direction. The signaling pathways that demonstrated the most conservancy between diet and gender included those involved in energy regulation (etc, bile acid, glycolysis), immune activity (IL-17) and, perhaps most interestingly, growth regulation/nutrient sensation (IGF1-mTOR). The IGF1-mTOR pathway demonstrated a very coherent level of regulation among the dietary groups of both genders, *i.e.* this pathway was significantly up-regulated in the same dietary groups (20% CR, 40% CR, HFG) in both genders. The random occurrence of this complex pathway regulation in males and females is highly unlikely and perhaps underlines a commonality of connectivity between the mTOR pathway, dietary energy consumption and gonadal transcription.

### Gene set to signaling pathway correlation in males and females

**Figure 15:**
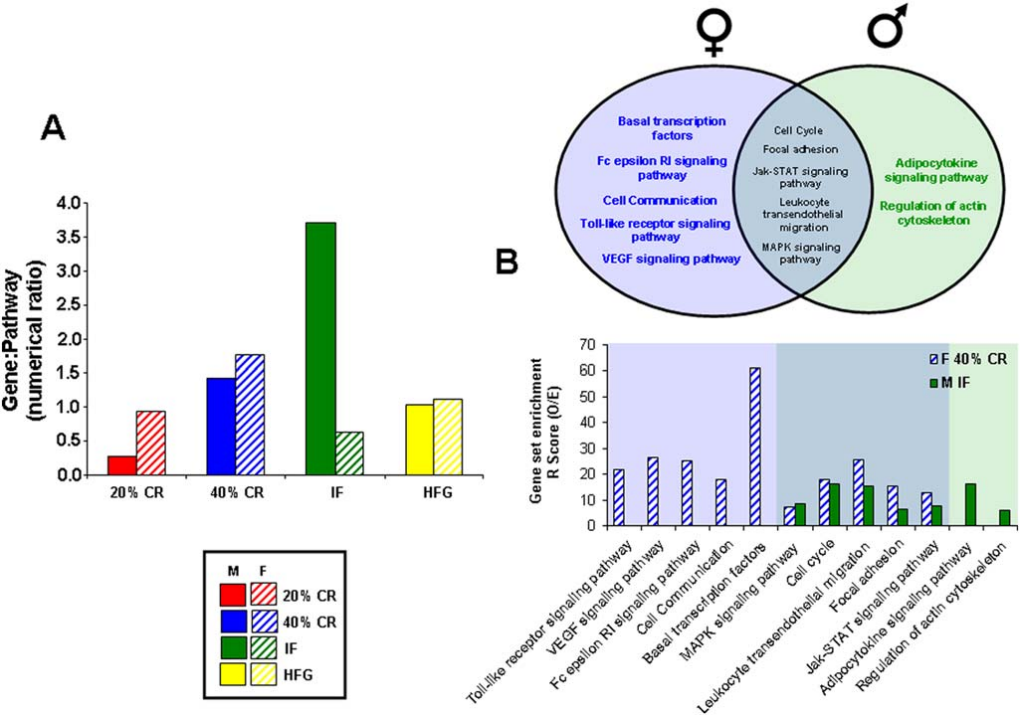
Correlation of genetic output between complementary animal responses to different dietary paradigms. Panel A depicts the gene∶pathway ratios created by each gender in response to the imposed dietary regime. Panel B demonstrates the functional cross-over between the KEGG functional pathway output of male IF compared to female 40% CR paradigms.

A simple index of the correlation between the linkage of individually-regulated genes in a gene set and their potential physiological function may provide an indication of the genetic and perhaps evolutionary coherency of an animal's response to its environment. All species have likely created ‘genetic programs’ that facilitate a rapid, coherent and specific genetic output in response to an environmental cue. The early generation of chemotactic responses, such as that of the slime molds, may be one of the earliest examples of this [9]. To investigate how individual gene responses relate to their potential phenotypic outcome, we created a numerical ratio between the number of statistically significantly regulated genes and the signaling pathways that these genes then significantly populate, when classified using an unbiased parametric gene set analysis method. When the ratio of significantly regulated genes to significantly populated signaling pathways was assessed for both genders across all dietary regimes (Figure 15A), it was clear that the 20% CR, 40% CR and HFG diets yielded similar gene∶pathway ratios. However, for the IF paradigm, the males demonstrated a considerably greater ratio of genes∶pathways. This increased ratio is indicative of the diet inducing a high degree of genetic transcriptional regulation, which eventually would create a relatively coherent physiological output. It is interesting to note that the 20% CR males, which received the same average caloric energy input, possessed a gene∶pathway ratio almost ten-fold lower than the IF males. These two male groups also possessed a similar end-study total body mass. Therefore such differences in gene coherency do not appear to be due to caloric input. Compared to the females, there appeared to be a distinction between the males' response between 20% CR and IF paradigms, *i.e.* in females there were no specific differences in the T/E ratio or the gene∶pathway ratio between female IF and 20% CR groups (Figures 2, 15). With respect to the genetic regulatory output, this distinction between male and female responses to IF when compared to 20% CR (negating any effect of caloric intake or body mass) was also apparent.

There were 126 significantly regulated genes different between IF and 20% CR groups in the males whereas there were only 57 significantly regulated genes different between IF and 20% CR females. When these genes, distinct between IF and 20% CR male or female groups, were objectively clustered according to their GO term annotations (performed using WebGestalt: http://bioinfo.vanderbilt.edu/webgestalt/) there were also more significantly populated GO term groups in males (23) compared to the females (8: Table 1). Genetic clustering of these specific gene sets with KEGG (Kyoto Encycolpedia of Genes and Genomes) signaling pathway annotation also revealed more significantly populated pathways for the males (6) compared to the females (4). It seems therefore that males possess a considerably more robust response specifically to IF compared to females. The phenotype of the calorie-independent IF-specific genes in the males contained the following GO term motifs: steroidal function (*cholesterol metabolism*); intracellular vesicle dynamics (*intracellular transport*, *microtubule cytoskeleton organization*, *regulation of endocytosis*); regulation of protein catabolism/degradation (*proteolysis during cellular protein catabolism*, *ubiquitin-dependent protein catabolism*, *protein ubiquitination*, *ubiquitin cycle*, *small conjugating protein-specific protease activity*, *cysteine-type endopeptidase activity*); cell signaling activity (*signal transduction*, *amino acid phosphorylation*, *ATP binding*, *Ser/Thr kinase activity*, *Tyr kinase activity*); transcriptional/translational regulation (*RNA polymerase II activity*, *nuclear mRNA splicing*, *transcriptional coactivator activity*, *regulation of translation*); stress management (*response to stress*). None of these GO term groups were present in the analysis of the calorie-independent IF genes in the corresponding female set (Table 1). The KEGG signaling pathway analysis also revealed that the male and female calorie-independent responses to IF were significantly different (Table 2). There were no common genes (data not shown) regulated in the male or female IF groups (when compared to 20% CR groups as a control to ensure calorie independence), no common GO term groups and no common KEGG signaling pathways. Thus the IF males possessed a unique response to the every other day absence of food.

**Table 1:** Webgestalt GO term analysis of gonadal transcriptional response to intermittent fasting compared to 20% caloric restriction.

MALE			FEMALE		
GO term	R	P	GO term	R	P
Signal transduction	6.25	4.08e-2	Amine metabolism	4.69	2.5e-2
Regulation of cell growth	6.25	4.16e-2	Smooth muscle differentiation	200	7.0e-5
Intracellular transport	2.47	3.30e-2	Mitosis	7.69	2.71e-2
Microtubule cytoskeleton organization	9.52	1.83e-2	Regulation of apoptosis	7.41	2.86e-2
Cholesterol metabolism	10.53	1.60e-2	Ribosome biogenesis	12	1.85e-3
Regulation of translation	8	2.59e-2	Chromatin remodeling	25	2.63e-3
Proteolysis during cellular protein catabolism	8.62	2.68e-4	Carbohydrate biosynthesis	20	4.48e-4
Ubiquitin-dependent protein catabolism	10.2	1.28e-4	Vessel development	6.25	4.02e-2
Amino acid phosphorylation	2.79	1.96e-2			
Protein ubiquitination	11.76	1.19e-2			
Ubiquitin cycle	4.31	5.82e-3			
RNA polymerase II activity	5.26	1.96e-2			
Nuclear mRNA splicing	8.89	1.02e-3			
Regulation of endocytosis	15.38	7.47e-3			
Response to stress	2.4	3.74e-2			
ATP binding	2.4	3.38e-3			
Transcription coactivator activity	6.9	3.39e-2			
Cysteine-type endopeptidase activity	5.66	1.57e-2			
Small conjugating protein-specific protease activity	11.54	2.31e-3			
Acetyltransferase activity	9.09	2.01e-2			
Ser/Thr kinase activity	15.38	7.21e-3			
Tyrosine kinase activity	3.42	2.96e-2			
Microsome activity	6.25	1.25e-2			

The genes significantly regulated in response to IF, but not 20% CR, for both genders were subjected to parametric Webgestalt GO term clustering. The enrichment factor [R] of the GO term groups is calculated by division of the observed (O) number of genes in the particular GO term group found in the experimental gene set by the expected (E) number of genes found in that set based on their background expression frequency in the Webgestalt genome set (R = O/E). The probability [P] stated is the probability of the specific degree of gene set enrichment [R] in the GO term group.

**Table 2:** Webgestalt KEGG pathway analysis of gonadal transcriptional response to intermittent fasting compared to 20% caloric restriction.

MALE			FEMALE		
KEGG pathway	R	P	KEGG pathway	R	P
Proteasome	44.84	9.21e-4	Ribosome	43.73	4.54e-5
Antigen processing and presentation	18.05	5.55e-3	Insulin signaling pathway	18.57	5.24e-3
Leukocyte transendothelial migration	11.72	1.28e-2	mTOR signaling	51.02	7.14e-4
MAPK signaling pathway	7.33	8.08e-3	Hedgehog signaling pathway	46.18	8.7e-4
Wnt signaling pathway	9.09	2.06e-2			
Regulation of actin cytoskeleton	6.70	3.6e-2			

The genes significantly regulated in response to IF, but not 20% CR, for both genders were subjected to parametric Webgestalt KEGG pathway clustering. The enrichment factor [R] of the GO term groups is calculated by division of the observed (O) number of genes in the particular KEGG pathway found in the experimental gene set by the expected (E) number of genes found in that set based on their background expression frequency in the Webgestalt genome set (R = O/E). The probability [P] stated is the probability of the specific degree of gene set enrichment [R] in the KEGG pathway group.

As we have proposed that there should be a complementarity of genetic regulation in gonadal transcriptional activity in species that reproduce through sexual reproduction, we investigated the females with the most similar physiological profile to the IF males. Among the female groups, the highest gene∶pathway ratio was observed in the 40% CR group. These 40% CR females had significantly reduced reproductive function and showed the lowest end-study body mass [1]. The female 40% CR rats' poor fertility status was also confirmed by their high, near male, T/E ratio (Figure 2). As we have shown, the male group with the highest T/E and gene∶pathway ratios was the male IF group. These findings may suggest that there could be a relationship between the transcriptional coherency of the male IF group and their T/E ratio. As the female group most similar to the male IF group in these respects was the 40% CR, we decided to further analyze their relationship using WebGestalt parametric analysis.

In order to compare the gonadal gene changes between the IF males and the 40% CR females, we used WebGestalt algorithms to group the significantly altered genes from the two diet paradigms into KEGG Pathways. There was a considerable degree of overlap between the two diets (Figure 15B). The 40% CR females had 10 significantly altered KEGG pathways, 5 of which were common with the IF males. In addition to the 5 shared KEGG pathways, the IF males had 2 unique KEGG pathways that were significantly altered (7 total). The overlapping KEGG pathway groups possessed a strong signaling phenotype as typified by the Jak-STAT and MAPK signaling pathways. This strong degree of transcriptional overlap could potentially underlie a gender-specific connection between the two sets of animals' differential response of males to every other day feeding and to extreme caloric restriction (40% CR) in the females. With respect to gonadal transcriptional responses it is likely, due to the need for sexual reproduction to be successful to propagate a species, that coherent ‘genetic programs’ are expressed complementarily in males and females in similar environments.

## Discussion

We investigated the overall gonadal transcriptional responses in adult male and female rats to different dietary paradigms including dietary restriction, every other day feeding and caloric excess. We found that multiple and complex patterns of gene regulation were induced in both the genders in response to the disruption of their food supply. With respect to gonadal structure and function and the nature of the imposed diet, we noted a much greater variation in total testicular weight in response to the alterations in energy intake than in total ovarian weight (Figure 2). Complex endocrine feedback systems, such as the reproductive system, maintain a tightly-controlled homeostasis within that system. Hormones within the reproductive system function in concert and their ultimate physiological function will be determined by their relative plasma ratios to each other. Testosterone can typically function as an antagonist of estrogen, opposing many of estrogen's actions. Thus determining the ratio of testosterone to estrogen in plasma is a highly informative method of assessing endocrine homeostasis and functional output within the reproductive system. It has also been clinically demonstrated that through the accurate measurement of specific sex steroid hormone ratios, and not through individual hormone measurements, reliable prognostic data can be generated that correlates with the incidence of multiple pathophysiological or disease states such as male breast cancer [10], hyperthyroidism [11], diabetes [12], [13], cirrhosis of the liver [14], gall stones [14] and bone fractures [15], [16]. Therefore due to the physiological antagonistic activities of testosterone and estrogen, consideration of the relative levels of these potent hormones in animal models or patients is likely to yield more physiologically relevant data.

A detailed overview of the absolute plasma levels of some of the hormones involved in the major endocrine axes has been provided previously [1]. With respect to the steroidal hormone status of the animals, in males the majority of the diets induced considerable alterations in the circulating T/E ratios, while only the 40% CR diet induced a significant alteration in the T/E ratio in the females. This potentially suggests that the IF males had ‘hypermasculinized’, along with the strong masculinization of the 40% CR females. It is important to note that despite the same effective input calories, the 20% CR and IF males possessed unique gonadal transcriptome responses to the different dietary paradigms. This however was not observed in the females. This suggests that in males there was a specific calorie-independent response to the alternate day absence of available food energy.

At the level of specific gene regulation, the females tended to show a greater calorie-specificity in their gene responsiveness in the gonads, reminiscent to what we have previously shown in the hippocampus [2]. Males on the other hand did not seem to be as calorie sensitive in either tissue (Figure 7).

There were several cross-diet genes identified in the female gene sets. The gene Irf1 (interferon regulatory factor 1) was down-regulated in the ovaries of 20% CR and 40% CR females (Figure 7). This gene has been shown to be expressed in the human uterus during the mid to late secretory phase of the menstrual cycle, and its expression is regulated by the hormone prolactin [17]. In the mouse, Irf1 has been shown to be involved in the remodeling of uterine spinal arteries, which supply blood to the endometrium of the uterus during the luteal phase of the menstrual cycle, and the remodeling of these arteries is a requirement for successful implantation of the embryo during pregnancy [18]. Hspa8 (heat shock protein 70 kDa) was down- regulated in the 40% CR and IF ovaries. This gene has been shown to be sharply increased in the human endometrium after ovulation and is thought to play a protective role during implantation [19]. Gdf9 (growth differentiation factor 9) was increased in the 20% CR and HFG ovaries. This gene is an oocyte secreted paracrine factor essential for mammalian ovarian folliculogenesis [20]. Gdf-9 is co-expressed with BMP15 and these genes work together in the ovary to aid in the differentiation and development of the granulosa cells. Granulosa cells produce steroids and growth factors that aid in the development of the oocyte. Follicle-stimulating hormone (FSH) stimulates granulosa cells to convert androgens to estradiol, and after ovulation granulosa cells then produce progesterone [21].

Among the multi-diet regulated genes in the males, several are of note with respect to gonadal function. Nuclear RNA export factor 1 (Nxf1) was significantly down-regulated in both the 40% CR and IF paradigms. Nxf1 is also classified as the TIP-(tyrosine kinase interacting protein) associated protein (TAP). Nxf1 expression is tightly controlled, as it has been shown to be crucially involved in the normal development of both male neurons and germ cells in cases of Fragile X syndrome [22]. The cold shock gene, Csda, was specifically up-regulated across two diets that were typified by extremes of energy balance (40% CR and HFG). These cold-shock proteins have been shown to be crucial for the protection and development of spermatocytes and seminiferous tubules [23]. The gene Ncl (nucleolin) was also up-regulated by two diets (IF, HFG) and has been shown to be highly indicative of rapidly growing cells, as it is important for pre-mRNA processing and potential tumor formation [24]. Indicative of the increased functional activity in the male testes in response to dietary alteration (IF and HFG), there was a significant up-regulation of Nek7 (NIMA-related kinase 7). The Nek7 gene has recently been identified in the testes [25] where it is likely to be a prime controller of cell cycle progression and mitotic spindle assembly [26]. To facilitate additional cell growth/function in response to dietary alterations, it is often necessary to re-model the structure of an organ or tissue. A common series of proteins involved in such processes are metalloproteinases. With respect to gonadal function, Adamts19 (a disintegrin and metalloproteinase domain, with thrombospondin type-1 modules), significantly up-regulated in 20% CR, 40% CR and HFG diets, has been shown to be expressed in multiple tissues, and in particular the testes [27], [28]. The down-regulation of genes involved in apoptotic mechanisms would be expected in situations of enhanced cell growth and development, for example in the male IF and HFG paradigms, there was a significant down-regulation of Septin 10. Septins are a highly conserved family of GTP-binding cytoskeletal proteins implicated in apoptosis, cell cycle regulation and oncogenesis [29].

Taking into account the alterations in gonadal function and gene expression, it appears that males respond in a more reactive manner to the imposition of the various dietary regimes. When considering genes regulated across multiple dietary paradigms, the males showed a considerably greater degree of up-regulated genes compared to the females (Figure 7). This again was demonstrated when the functional signaling pathways were analyzed (Figure 13). This is perhaps not surprising when the distinct activities of the two genders' gonads are considered. Male spermatogenesis is a relatively robust activity that is maintained for a long period of time in a mammal's lifespan. Females on the other hand, are typically born with a set number of ova that then progressively decrease with time. The activity of the ovary is considerably more complex as it has to be timed to the menstrual cycle. Cyclicity in females is a sensitive phenomenon and can be dramatically affected by dietary perturbations. We have previously shown that female reproductive cycling capacity was considerably affected during the imposition of some of these dietary regimens [1]. Even small alterations in input energy caused the females to largely cease cycling. Many previous studies have demonstrated the relative robustness of continued spermatogenesis in the face of dietary alteration in multiple species [30], [31]. It is interesting to note however that in our transcriptional analysis there seemed to be a stronger up-regulating response in the males compared to the females, despite the history of male resilience to environmental dietary alteration. With respect to this observation, perhaps the most important finding was the unique nature of the males' response to the every other day feeding regime. When the relationship between their transcriptional response to IF was compared to the eventual physiological phenotype this may induce, we found that this gene∶pathway ratio was extremely high for the male IF group (Figure 15). Comparing this response to the 20% CR males, we are able to conclude that this effect is independent of the input calories to these two groups [1]. This calorie-independent response seemed unique to the male IF group, as the female IF group gene∶pathway ratio was considerably lower. When the genetic output (genes, GO terms and KEGG pathways) of the male or female IF groups were both compared to their corresponding 20% CR groups, there was also a large difference in the male IF response compared to the females (Tables 1, 2). This may indicate that the males possessed a specific response to the periodic absence of food. Even the presence of low levels of food (40% CR) did not induce this high gene∶pathway ratio. The male IF group also showed the highest circulating T/E ratio and the largest degree of change in testes mass (Figure 2). Leptin and growth hormone were each significantly decreased in the IF males as were plasma corticosterone levels, though not statistically significantly [1]. These hormonal changes caused by the IF diet are reflective of a tendency towards ‘heightened’ masculinity.

As we have postulated, alterations in the reproductive status of animals that reproduce sexually are likely to occur in a complementary manner. In an environment temporarily devoid of available food, *i.e.* the IF diet, the male would assume that females also will experience this environment and would therefore have a ‘predictable’ lower degree of fertility, as the females possess a lower capacity to compete for food than the males. Hence for the species to propagate, the male would need to increase the chance that an encounter with a female would result in successful fertilization; and therefore, the male needs a heightened degree of fertility. In the male IF group the increased testes mass, elevated circulating T/E ratio and the highest genetic output coherency all suggest a pre-programmed physiological response specific to periods of food absence, and not food paucity. If the male ‘predicts’ the presence of semi-starved females (*i.e.* the male has no access to food and therefore the poorly-competing female may have even less), then it is likely that there would be a complementarity in gonadal genetic regulation between the two genders. Thus, we would expect a similarity in genetic output between the IF male and the most malnourished female, *i.e.* the 40% CR group. Performing this analysis, we noted a significant degree of identity between the significantly regulated KEGG signaling pathways between the male IF group and the female 40% CR group (Figure 15). It is also interesting to note that there was a hormonal link between these two groups, as they both possessed the highest circulating T/E ratios. Also linking the male IF group to the 40% CR female group, the 40% CR females showed the greatest gene∶pathway coherency amongst the female dietary paradigms (Figure 15). Similarly to the IF males, the 40% CR females had significantly lower plasma leptin levels than the *ad libitum* controls [1]. Conversely, though, the 40% CR females had similar growth hormone levels to controls. Corticosterone levels were significantly increased, reflecting the extreme stress the 40% CR diet placed on the female rats [1].

Interestingly, the 40% CR females showed a significant down-regulation in the gene Ccnd2 (cyclin D2), which is involved in the focal adhesion, JAK-STAT signaling, and cell cycle KEGG pathways. In the ovary, ccnd2 is expressed in the granulosa cells of the follicles and has been shown to play an important role in folliculogenesis. Ccnd2 null mice have been shown to be infertile due to impaired granulosa cell proliferation in response to follicle stimulating hormone (FSH) and thus have small follicles with impaired oocyte release [32], [33]. Thus, this reduction in ccnd2 may be reflective of the impaired reproductive cycling ability of these rats. The 40% CR females showed a down-regulation in two genes involved in the JAK-STAT signaling pathway, which has been shown to play an important role in the maintenance of germline stem cells and follicle development in the drosophila ovary and testis [34]–[36]. The 40% CR females also showed a down-regulation in the vascular endothelial growth factor (VEGF) signaling pathway, which plays an essential role in angiogenesis and follicle development within the female reproductive tract [37]–[39]. The IF males also showed several interesting gene changes correlated to the KEGG pathway alterations. There was a significant increase in the Fgf10 (fibroblast growth factor 10) gene, which is involved in the MAPK signaling pathway and has shown to play a role in the growth and development of several reproductive tissues [40]. Pcna (proliferating cell nuclear antigen), which is a part of the cell cycle KEGG pathway, was significantly down-regulated in the IF testes. Pcna is highly expressed in the sertoli cells of the testes, which function to nurture developing sperm cells during spermatogenesis [41] and is often used as a marker for cell proliferation.

Genes with differential expression patterns in the male and female rats, that were associated with fertility and reproductive tract morphology and function, were identified and then subjected to unbiased bioinformatic analysis. Estimation of the potential functional relevance of these genes was performed using the Jackson Lab Mouse Genome Database. This resource provides data correlating specific gene alterations and multiple physiological or pathophysiological outcomes (Table 3). In the IF males, various fertility related genes were up-regulated compared to the control males, such as Cugbp1 (Cug-binding protein), Gnpat (Glyceronephosphate o-acyltransferase), Smtn (Smoothelin), Fgf10 (Fibroblast growth factor 10). These genes play important roles in the maintenance of spermatogenesis and seminiferous tubule morphology [42]–[45]. In the 40% CR males, there was an up-regulation of Csda (Cold-shock domain protein A), Ube3a (Ubiquitin-protein ligase E3A), and Col4a1 (Collagen type IV). These genes are involved in maintaining spermatogenesis, seminiferous tubule structure, and fertility [23], [46], [47]. The up-regulation of these genes in the male IF and 40% CR animals suggests that these male animals are potentially maintaining or up-regulating their reproductive ability during periods of food scarcity. In the 20% CR female animals, there was an up-regulation in Hsd17b4 (17-beta-hydroxysteroid dehydrogenase IV), Gdf9 (Growth/differentiation factor 9), and Ccnd2 (Cyclin D2). These are important factors for maintaining ovarian morphology, folliculogenesis, and ovulation [48]–[50]. This suggests that the 20% CR females are potentially maintaining their reproductive ability, despite having reduced caloric input compared to the control females. The 40% CR females on the other hand, show a decrease in Ccnd2 expression. This could potentially suggest that ovulatory function is impaired in these severely calorie-restricted females and that they exhibit reduced reproductive ability compared to the control female rats.

**Table 3:** Alterations in fertility-related genes in the male and female rats on the different dietary regimes.

Gene symbol	Gene name	Knock-out mouse phenotype	Alteration in diet group	Up- or down-regulated
Cugbp1	Cug-binding protein	Arrest of spermatogenesis, reduced male and female fertility, abnormal testicular physiology	Male IF	↑
Gnpat	Glyceronephosphate o-acyltransferase	Small ovaries, male infertility, abnormal seminiferous tubule morphology, azoospermia, arrest of male meiosis	Male IF	↑
Smtn	Smoothelin	Infertility	Male IF	↑
Fgf10	Fibroblast growth factor 10	Abnormal male reproductive anatomy, abnormal prostate morphology	Male IF	↑
Hsd17b4	17-beta-hydroxysteroid dehydrogenase IV	Alterations in reproductive function, reduced male fertility	Female 20% CR	↑
Gdf9	Growth/differentiation factor 9	Abnormal ovarian morphology, abnormal folliculogenesis, increased circulating follicle stimulating hormone, increased circulating luteinizing hormone, female infertility	Female 20% CR	↑
			Female HFG	↑
Ccnd2	Cyclin D2	Abnormal ovulation, oligozoospermia, female infertility	Female 20% CR	↑
			Female 40% CR	↓
Csda	Cold-shock domain protein A	Reduced testis size, seminiferous tubule degeneration, abnormal spermatogenesis, male infertility	Male 40% CR	↑
			Male HFG	↑
Ube3a	Ubiquitin-protein ligase E3A	Reduced male fertility, decreased testis weight, reduced spermatogenesis, decreased prostate weight, abnormal ovarian folliculogenesis, reduced female fertility	Male 40% CR	↑
Col4a1	Collagen type IV	Reduced fertility	Male 40% CR	↑

Genes with differential expression in the male and female rats, that were associated with fertility and reproductive tract morphology and function, were functionally analyzed using the Jackson Labs Mouse Genome Database.

Taken together, these findings suggest the presence of a pre-programmed genetic response in males to periodic food absence that in a sense ‘predicts’ the fecundity of its potential mates and adjusts reproductive function accordingly. Interestingly, this phenomenon still takes place in the absence of physical contact between the two genders. The male therefore, with its robust reproductive capacity, may serve as a functional ‘rheostat’ for the species' reproduction, linking temporal food availability to the propagation of the species.

## Materials and Methods

### Animals and diets

Forty seven male and forty seven female Sprague-Dawley rats were singly housed on a 12 hr light/dark cycle. The following diets were applied to the rats beginning at 4 months of age: control (*ad libitum*); 20% CR, 40% CR; IF (alternate day fasting); and HFG. Control, CR and IF groups received food pellets that contained 19% protein, 64% carbohydrates, and 17% fat (diet 101845 from Dyets Inc., Bethlehem, PA); this food had a caloric density of 3.774 cal/g and a glycemic load/kg of 442. The HFG diet (diet 101842 from Dyets Inc.) contained 15% protein, 38% carbohydrates, and 47% fat. The caloric density of the HFG diet was 4.645 cal/g and its glycemic load/kg was 363. Weights were recorded for each rat on a regular basis throughout the study. The reproductive status was monitored closely for the female rats using cervical smear analysis. All the female rats were euthanized when they were estimated to be in the diestrous stage of their reproductive cycle. All procedures were performed in accordance with approved institutional protocols and were approved by the Institutional Animal Care and Use Committee of the National Institute on Aging.

### Tissue, plasma collection and plasma analyses

At the end of the study, the 10-month-old rats were euthanized using isoflurane anesthesia followed by decapitation. Upon euthanasia, the gonads were carefully dissected out. Tissues were flash frozen on dry ice and stored at −80°C until further analyses. Plasma estradiol and testosterone levels were measured using RIA by Dr A.F. Parlow, National Hormone and Peptide Program, Torrance, CA.

### RNA extraction

The gonadal tissue was processed using a Bead Beater (Bio-Spec, Bartlesville, OK) followed by RNA purification using the RNEasy Mini Kit (Qiagen, Valencia, CA) according to the manufacturer's instructions. The RNA was examined for quantity and quality using an Agilent Bioanalyzer 2100 (Agilent Technologies, Palo Alto, CA).

### Radioactive cDNA probe preparation and microarray hybridization

cDNA probe preparation and microarray hybridization were performed as described previously [51]. Briefly, 5 µg total RNA was reverse-transcribed in a reaction mixture containing 8 µl of 5× first strand RT buffer, 1 µl of 1 µg/µl 12–18 mer poly (dT) primer, 4 µl of 20 mM dNTPs (-dCTP), 4 µl of 0.1 M DTT, 1 µl (40 U) of RNaseOUT, 6 µl of 3000 Ci/mmol α-^33^P-dCTP and DEPC-water to a final volume of 40 µl. The RT mixture was first heated at 65°C for 10 min, followed by incubation on ice for 2 min. Two microliters of Superscript II reverse transcriptase (Life Technologies, CA) was then added followed by incubation at 42°C for 35 min. One additional microliter of reverse transcriptase was added, followed by another 35 minute incubation. At the end of the incubation, 5 µl of 0.5 M EDTA was added to chelate divalent cations. After addition of 10 µl of 1.0 M NaOH, the samples were incubated at 65°C for 30 min to hydrolyze the remaining RNA. Following the addition of 25 µl of 1 M Tris (pH 8.0), the samples were purified using Bio-Rad 6 purification columns (Hercules, CA). cDNA microarrays were pre-hybridized in a 4 ml hybridization buffer containing 3.2 ml Microhyb (Research Genetics, AL) and 0.8 ml 50% dextran sulfate, 10 µl of 10 mg/ml denatured human Cot 1 DNA (Life Technologies) and 10 µl of 8 mg/ml denatured poly(dA) (Pharmacia, NJ). After at least 4 h of pre-hybridization at 55°C, approximately 10^6^ cpm/ml of heat-denatured cDNA probes were added, followed by 17 h of incubation at 55°C. Hybridized arrays were washed in 2× SSC and 0.1% SDS once at room temperature followed by two washes in 2× SSC and 0.1% SDS at 65°C for 15 min each.

### Scanning and quantification

The microarrays were exposed to phosphorimager screens for 3 days. The screens were then scanned in a Molecular Dynamics STORM PhosphorImager (Sunnyvale, CA) at 50 µm resolution. Quantification of scanned screens was performed with ArrayPro software.

### Z-scores and z-ratio

Raw hybridization intensity data were log-transformed and normalized to yield z-scores, which in turn were used to calculate a z-ratio value for each gene with respect to the control tissues. The z-ratio was calculated as the difference between the observed gene z-scores for the experimental and the control comparisons, and dividing by the standard deviation associated with the distribution of these differences [52]. Z-ratio values ≥+2.0 or ≤−2.0 were chosen as cut-off values, defining increased and decreased expression, respectively.

### Filtering and cluster analysis

DIANE (NIH) software was used to filter the 17,000 genes. We filtered out genes which did not vary at least 1.25-fold from the log of the mean of the first filter in at least 60% of the genes expressed (p<0.01). Genes were clustered and sub-clusters were generated using DIANE software.

### Venn diagram generation

Multiple Venn diagrams were constructed using an online generator (http://www.pangloss.com/seidel/Protocols/venn.cgi) to identify and isolate the genes that were either significantly up-regulated or significantly down-regulated compared to the *ad libitum* (control) rats. In addition to being significant at p<0.01, the changes needed to vary by greater than 25% from the controls using the median of the log value of the first filter.

### Gene pathway analyses

A complete set of 522 cellular pathways was obtained from the Molecular Signatures Database (MSigDB) created by the Broad Institute at the Massachusetts Institute of Technology [53]. The complete set was tested for Geneset enrichment using Parametric analysis of Gene set enrichment (PAGE, [54]). For each pathway a z-score was computed as previously described [2]. For each pathway z-score a p-value was computed using JMP 6.0 software to test for the significance of the z-score obtained. These tools were part of the DIANE 1.0 analytical software suite (http://www.grc.nia.nih.gov/branches/rrb/dna/diane_software.pdf for information). Additional parametric analysis to generate gene ontology (GO) term clusters and KEGG (Kyoto Encyclopedia of Genes and Genomes: http://www.genome.jp/kegg/) pathway clustering was performed using WebGesalt algorithms (http://bioinfo.vanderbilt.edu/webgestalt/).

### Functional gene analyses

Multiple genes with differential expression in the male and female rats, that were associated with fertility and reproductive tract morphology and function, were analyzed. A functional analysis was performed using the Jackson Labs Mouse Genome Database (http://www.informatics.jax.org/phenotypes.shtml). This Genome Database includes murine data on gene characterization, nomenclature, mapping, gene homologies, sequence links, phenotypes, allelic variants and mutants, and strain data [55].

## Supporting Information

Figure S1 Male multi-diet comparison genes. Significantly altered genes in the testes of the male rats placed on each of the four experimental diets (20% CR, 40% CR, IF, or HFG) were clustered into a 4-way Venn diagram. Letters A-O (seen in key) report the number of common gene alterations between the various diets. Specific gene names are reported in the columns at the top of the figure. Red genes were up-regulated and green genes were down-regulated. Names of the significantly altered genes can be found in Table S1.
(0.11 MB TIF)

Figure S2 Female multi-diet comparison genes. Significantly altered genes in the ovaries of the female rats placed on each of the four experimental diets (20% CR, 40% CR, IF, or HFG) were clustered into a 4-way Venn diagram. Letters A-O (seen in key) report the number of common gene alterations between the various diets. Specific gene names are reported in the columns at the top of the figure. Red genes were up-regulated and green genes were down-regulated. Names of the significantly altered genes can be found in Table S1.
(0.11 MB TIF)

Figure S3 Significant gene pathway changes in the gonads of male and female rats maintained on a 20% CR diet. Significantly altered genes in the male and female gonads from the different dietary regimes were clustered into functional gene pathways. In the testes from male rats on the 20% CR diet, there were 47 significantly altered gene pathways, of which 8 pathways were significantly down-regulated and 39 pathways were significantly up-regulated, compared to gene pathways in testes from male control rats. Interestingly, the ovaries from female rats on the 20% CR diet showed a very different functional gene pathway pattern as there were 50 significantly altered pathways, of which 30 were significantly down-regulated and 20 were significantly up-regulated, compared to gene pathways in ovaries from control female rats.
(0.10 MB TIF)

Figure S4 Significant gene pathway changes in the gonads of male and female rats maintained on a 40% CR diet. Significantly altered genes in the male and female gonads from the different dietary regimes were clustered into functional gene pathways. In the testes from male rats on the 40% CR diet, there were 42 significantly altered gene pathways, of which 17 pathways were significantly down-regulated and 25 pathways were significantly up-regulated, compared to gene pathways in testes from male control rats. The ovaries from female rats on the 40% CR diet showed a similar functional gene pathway pattern. There were 33 significantly altered pathways, of which 14 were significantly down-regulated and 19 were significantly up-regulated, compared to gene pathways in ovaries from control female rats.
(0.10 MB TIF)

Figure S5 Significant gene pathway changes in the gonads of male and female rats maintained on an IF diet. Significantly altered genes in the male and female gonads from the different dietary regimes were clustered into functional gene pathways. In the testes from male rats on the IF diet, there were 37 significantly altered gene pathways, of which 13 pathways were significantly down-regulated and 24 pathways were significantly up-regulated, compared to gene pathways in testes from male control rats. Interestingly, the ovaries from female rats on the IF diet showed a very different functional gene pathway pattern as there were 55 significantly altered pathways, of which 39 were significantly down-regulated and 16 were significantly up-regulated, compared to gene pathways in ovaries from control female rats.
(0.10 MB TIF)

Figure S6 Significant gene pathway changes in the gonads of male and female rats maintained on a HFG diet. Significantly altered genes in the male and female gonads from the different dietary regimes were clustered into functional gene pathways. In the testes from male rats on the HFG diet, there were 32 significantly altered gene pathways, of which 12 pathways were significantly down-regulated and 20 pathways were significantly up-regulated, compared to gene pathways in testes from male control rats. Interestingly, the ovaries from female rats on the HFG diet showed a very different functional gene pathway pattern as there were 47 significantly altered pathways, of which 30 were significantly down-regulated and 17 were significantly up-regulated, compared to gene pathways in ovaries from control female rats.
(0.10 MB TIF)

Figure S7 Male multi-diet comparison pathways. Significantly altered pathways in the testes of the male rats placed on each of the four experimental diets (20% CR, 40% CR, IF, or HFG) were clustered into a 4-way Venn diagram. Letters A-O (seen in key) report the number of common gene alterations between the various diets. Specific gene names are reported in the columns at the top of the figure. Red genes were up-regulated and green genes were down-regulated. One pathway, shh_lisa, was incoherently regulated between the 20% CR and 40% CR diets. This pathway was up-regulated in the gonads of the 20% CR males and down-regulated in the gonads of the 40% CR males. Names of the significantly altered pathways can be found in Table S2.
(0.09 MB TIF)

Figure S8 Female multi-diet comparison pathways. Significantly altered pathways in the ovaries of the female rats placed on each of the four experimental diets (20% CR, 40% CR, IF, or HFG) were clustered into a 4-way Venn diagram. Letters A-O (seen in key) report the number of common gene alterations between the various diets. Specific gene names are reported in the columns at the top of the figure. Red genes were up-regulated and green genes were down-regulated. One pathway, intrinsic, was incoherently regulated between the 20% CR and 40% CR diets. This pathway was up-regulated in the gonads of the 20% CR females and down-regulated in the gonads of the 40% CR females. Names of the significantly altered pathways can be found in Table S2.
(0.10 MB TIF)

Table S1 Gene symbols and gene names.
(0.37 MB DOC)

Table S2 PAGE pathway abbreviations: Molecular Signatures Database (www.broad.mit.edu/gsea/msigdb/genesets.jsp)
(0.41 MB DOC)
