# Supplementary figures and images for: Gonadal Transcriptome Alterations in Response to Dietary Energy Intake: Sensing the Reproductive Environment
Source: PLoS One. 2009 Jan 7;4(1):e4146. doi: 10.1371/journal.pone.0004146 (PMC2607546; doi:10.1371/journal.pone.0004146)

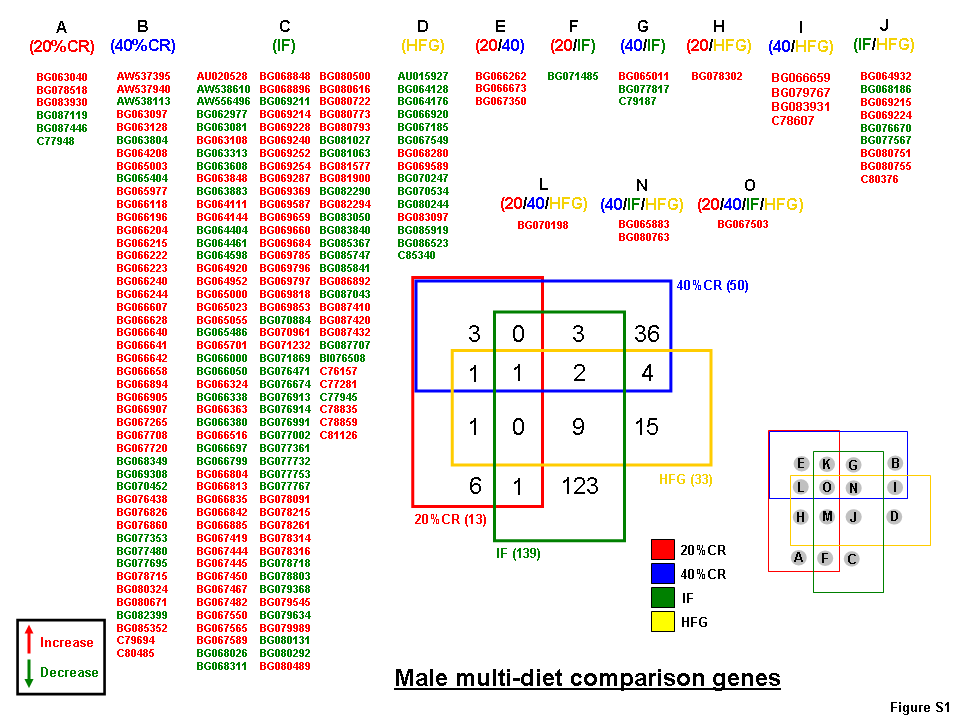

Supplement: Figure S1 — Male multi-diet comparison genes. Significantly altered genes in the testes of the male rats placed on each of the four experimental diets (20% CR, 40% CR, IF, or HFG) were clustered into a 4-way Venn diagram. Letters A-O (seen in key) report the number of common gene alterations between the various diets. Specific gene names are reported in the columns at the top of the figure. Red genes were up-regulated and green genes were down-regulated. Names of the significantly altered genes can be found in Table S1. (0.11 MB TIF) [file pone.0004146.s001.tif]

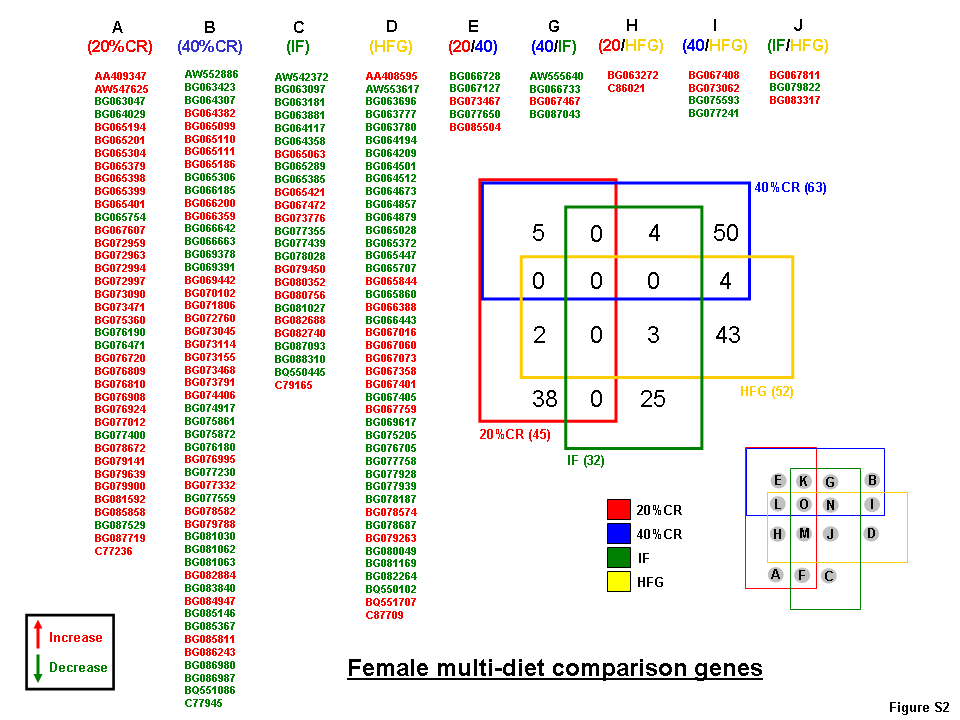

Supplement: Figure S2 — Female multi-diet comparison genes. Significantly altered genes in the ovaries of the female rats placed on each of the four experimental diets (20% CR, 40% CR, IF, or HFG) were clustered into a 4-way Venn diagram. Letters A-O (seen in key) report the number of common gene alterations between the various diets. Specific gene names are reported in the columns at the top of the figure. Red genes were up-regulated and green genes were down-regulated. Names of the significantly altered genes can be found in Table S1. (0.11 MB TIF) [file pone.0004146.s002.tif]

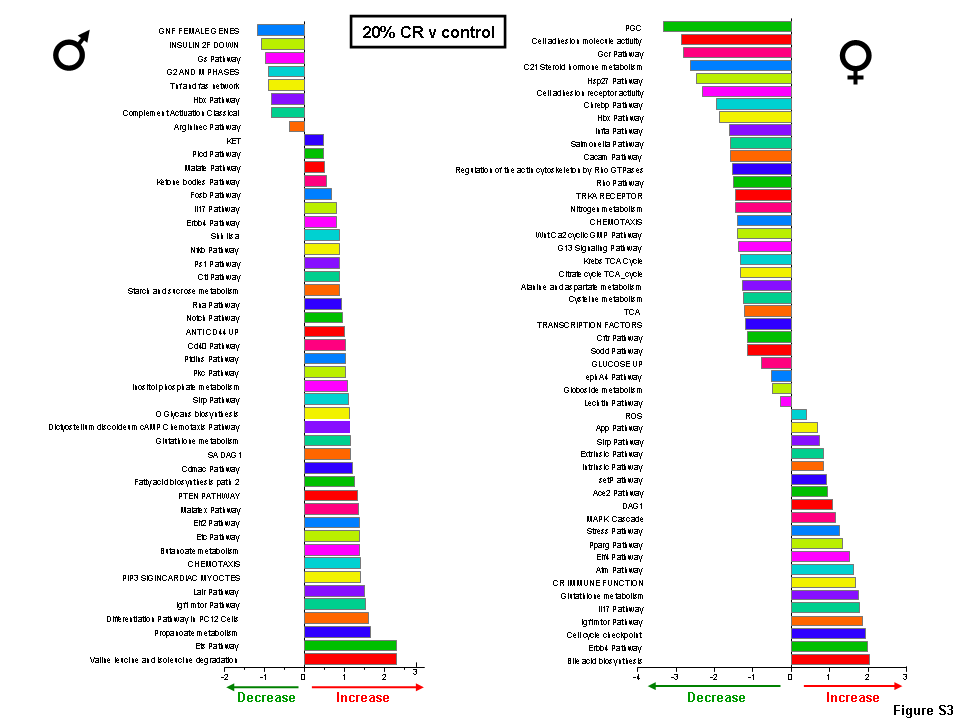

Supplement: Figure S3 — Significant gene pathway changes in the gonads of male and female rats maintained on a 20% CR diet. Significantly altered genes in the male and female gonads from the different dietary regimes were clustered into functional gene pathways. In the testes from male rats on the 20% CR diet, there were 47 significantly altered gene pathways, of which 8 pathways were significantly down-regulated and 39 pathways were significantly up-regulated, compared to gene pathways in testes from male control rats. Interestingly, the ovaries from female rats on the 20% CR diet showed a very different functional gene pathway pattern as there were 50 significantly altered pathways, of which 30 were significantly down-regulated and 20 were significantly up-regulated, compared to gene pathways in ovaries from control female rats. (0.10 MB TIF) [file pone.0004146.s003.tif]

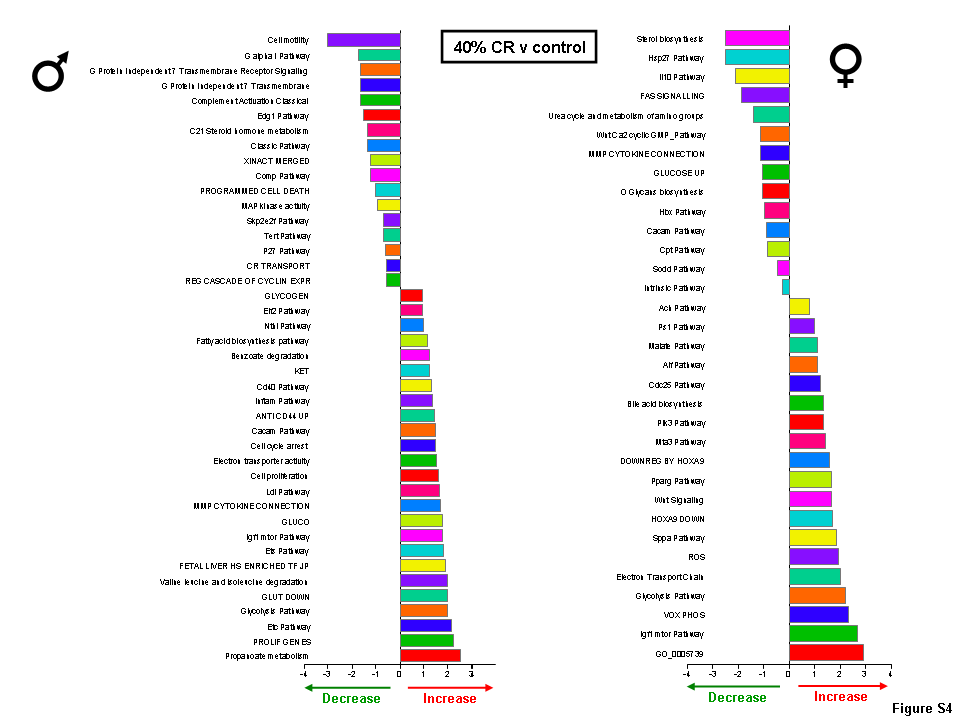

Supplement: Figure S4 — Significant gene pathway changes in the gonads of male and female rats maintained on a 40% CR diet. Significantly altered genes in the male and female gonads from the different dietary regimes were clustered into functional gene pathways. In the testes from male rats on the 40% CR diet, there were 42 significantly altered gene pathways, of which 17 pathways were significantly down-regulated and 25 pathways were significantly up-regulated, compared to gene pathways in testes from male control rats. The ovaries from female rats on the 40% CR diet showed a similar functional gene pathway pattern. There were 33 significantly altered pathways, of which 14 were significantly down-regulated and 19 were significantly up-regulated, compared to gene pathways in ovaries from control female rats. (0.10 MB TIF) [file pone.0004146.s004.tif]

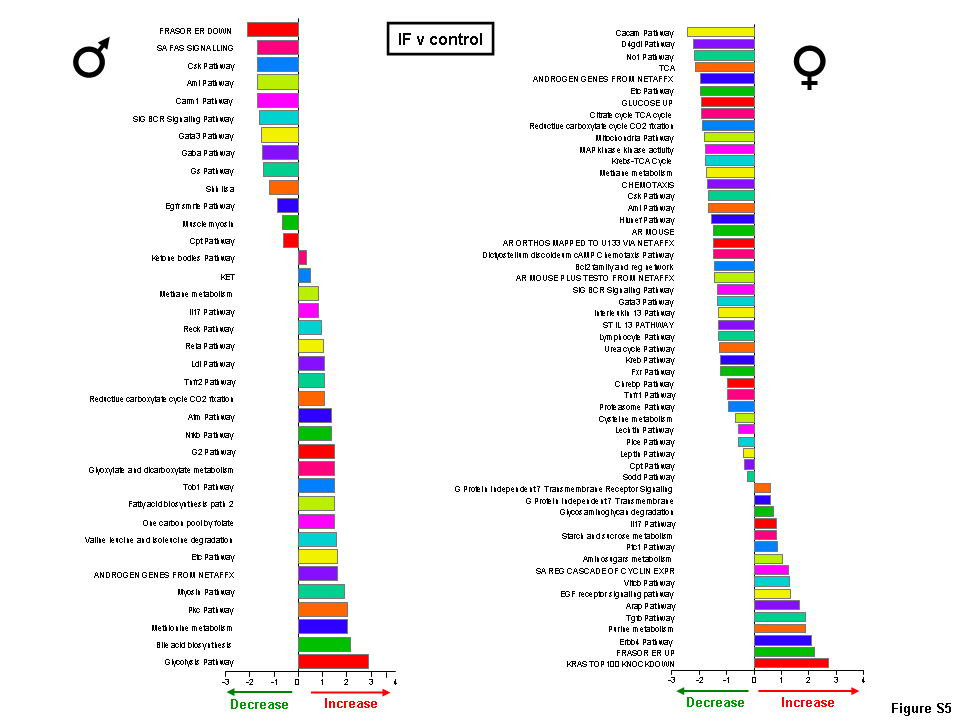

Supplement: Figure S5 — Significant gene pathway changes in the gonads of male and female rats maintained on an IF diet. Significantly altered genes in the male and female gonads from the different dietary regimes were clustered into functional gene pathways. In the testes from male rats on the IF diet, there were 37 significantly altered gene pathways, of which 13 pathways were significantly down-regulated and 24 pathways were significantly up-regulated, compared to gene pathways in testes from male control rats. Interestingly, the ovaries from female rats on the IF diet showed a very different functional gene pathway pattern as there were 55 significantly altered pathways, of which 39 were significantly down-regulated and 16 were significantly up-regulated, compared to gene pathways in ovaries from control female rats. (0.10 MB TIF) [file pone.0004146.s005.tif]

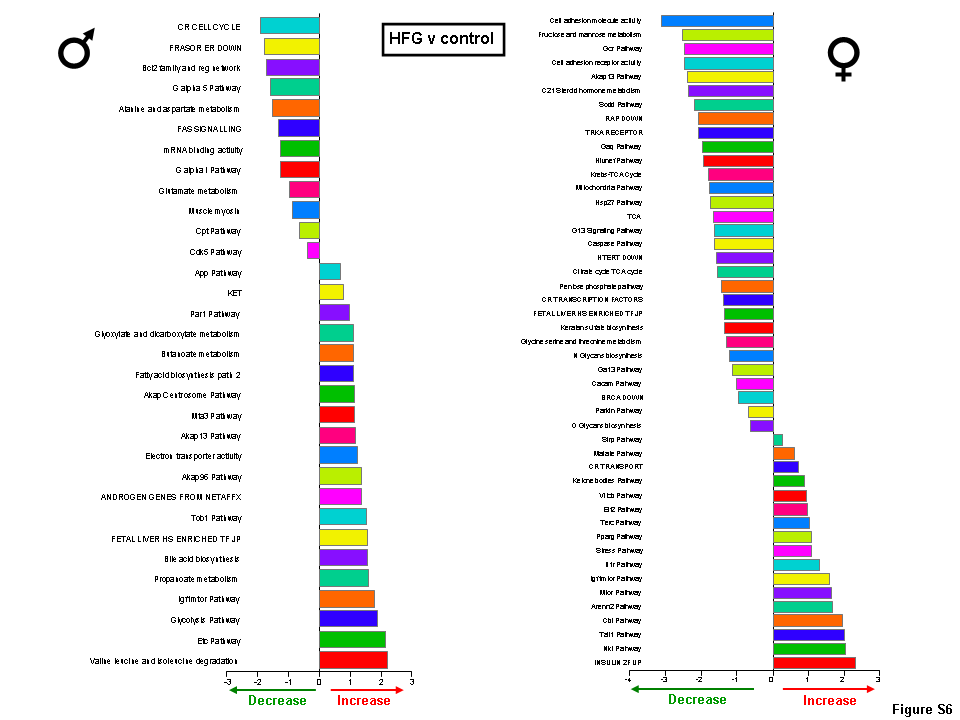

Supplement: Figure S6 — Significant gene pathway changes in the gonads of male and female rats maintained on a HFG diet. Significantly altered genes in the male and female gonads from the different dietary regimes were clustered into functional gene pathways. In the testes from male rats on the HFG diet, there were 32 significantly altered gene pathways, of which 12 pathways were significantly down-regulated and 20 pathways were significantly up-regulated, compared to gene pathways in testes from male control rats. Interestingly, the ovaries from female rats on the HFG diet showed a very different functional gene pathway pattern as there were 47 significantly altered pathways, of which 30 were significantly down-regulated and 17 were significantly up-regulated, compared to gene pathways in ovaries from control female rats. (0.10 MB TIF) [file pone.0004146.s006.tif]

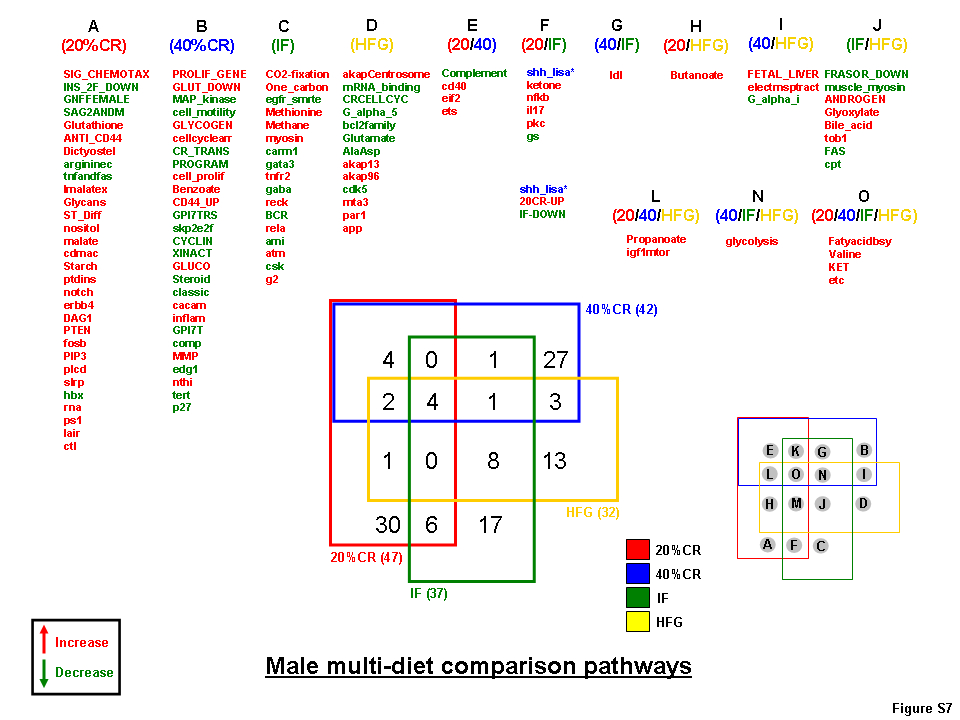

Supplement: Figure S7 — Male multi-diet comparison pathways. Significantly altered pathways in the testes of the male rats placed on each of the four experimental diets (20% CR, 40% CR, IF, or HFG) were clustered into a 4-way Venn diagram. Letters A-O (seen in key) report the number of common gene alterations between the various diets. Specific gene names are reported in the columns at the top of the figure. Red genes were up-regulated and green genes were down-regulated. One pathway, shh_lisa, was incoherently regulated between the 20% CR and 40% CR diets. This pathway was up-regulated in the gonads of the 20% CR males and down-regulated in the gonads of the 40% CR males. Names of the significantly altered pathways can be found in Table S2. (0.09 MB TIF) [file pone.0004146.s007.tif]

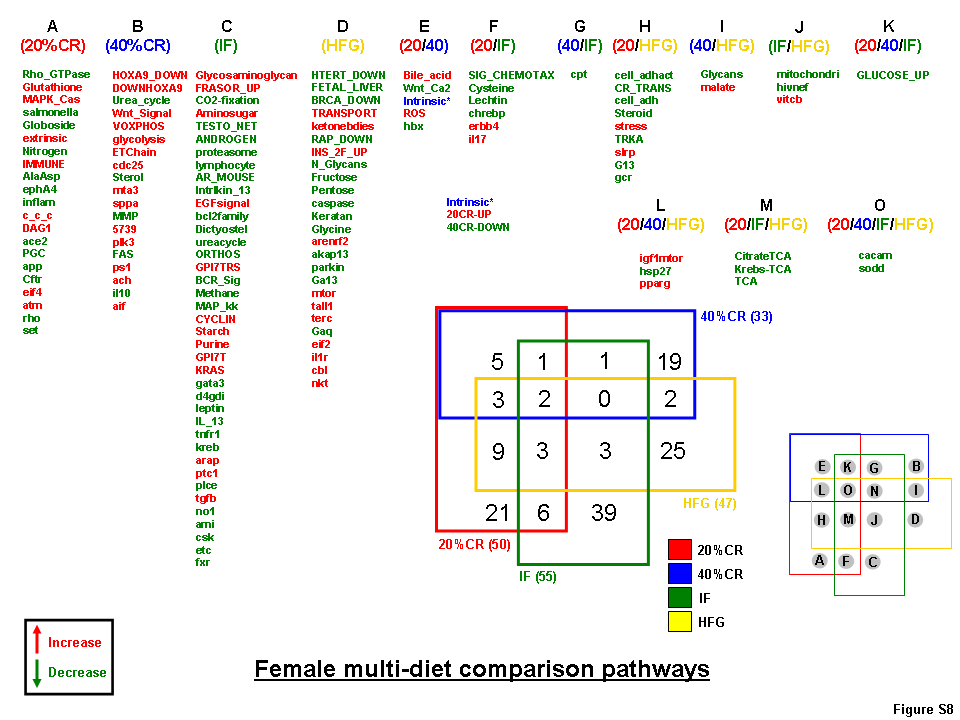

Supplement: Figure S8 — Female multi-diet comparison pathways. Significantly altered pathways in the ovaries of the female rats placed on each of the four experimental diets (20% CR, 40% CR, IF, or HFG) were clustered into a 4-way Venn diagram. Letters A-O (seen in key) report the number of common gene alterations between the various diets. Specific gene names are reported in the columns at the top of the figure. Red genes were up-regulated and green genes were down-regulated. One pathway, intrinsic, was incoherently regulated between the 20% CR and 40% CR diets. This pathway was up-regulated in the gonads of the 20% CR females and down-regulated in the gonads of the 40% CR females. Names of the significantly altered pathways can be found in Table S2. (0.10 MB TIF) [file pone.0004146.s008.tif]
