# Supplementary material for: Gonadal Transcriptome Alterations in Response to Dietary Energy Intake: Sensing the Reproductive Environment
Source: PLoS One. 2009 Jan 7;4(1):e4146. doi: 10.1371/journal.pone.0004146 (PMC2607546; doi:10.1371/journal.pone.0004146)
Supplement: Table S1 — Gene symbols and gene names. (0.37 MB DOC) [file pone.0004146.s009.doc]

**Table S1: Gene symbols and gene names.**

| **Gene Names** | **GB accession number** | **Symbol** |
| --- | --- | --- |
| 1-acylglycerol-3-phosphate O-acyltransferase 1 (lysophosphatidic acid acyltransferase, alpha) | BG077400 | Agpat1 |
| 3-phosphoglycerate dehydrogenase | BG066673 | Phgdh |
| actin, beta, cytoplasmic | C78835 | Actb |
| activator of S phase kinase | BG081169 | AA545217 |
| adaptor protein complex AP-2, alpha 1 subunit | BG077650 | Ap2a1 |
| adenylosuccinate lyase | BG076914 | Adsl |
| albumin 1 | BG079989 | Alb1 |
| aldo-keto reductase family 1, member C13 | BG082884 | Akr1c13 |
| aminolevulinate, delta-, dehydratase | BG080616 | Alad |
| AMP deaminase 3 | BG080773 | Ampd3 |
| ancient ubiquitous protein | BG079822 | Prss25 |
| apolipoprotein A-I | BG066443 | Apoa1 |
| aryl-hydrocarbon receptor | BG082290 | Usp47 |
| ATP synthase, H+ transporting, mitochondrial F0 complex, subunit c (subunit 9), isoform 1 | AW538610 | Atp5g1 |
| ATP synthase, H+ transporting, mitochondrial F0 complex, subunit f, isoform 2 | BG073062 | Atp5j2 |
| ATP-binding cassette, sub-family C (CFTR/MRP), member 3 | BG082294 | Abcc3 |
| autophagy 5-like (S. cerevisiae) | BG080763 | Apg5l |
| baculoviral IAP repeat-containing 2 | BG069214 | Birc2 |
| B-cell src-homology tyrosine kinase | BG067073 | Frk |
| Bcl2-interacting killer-like | BG066223 | Arpc2 |
| bisphosphate 3'-nucleotidase 1 | BG067708 | Bpnt1 |
| bromodomain-containing 2 | BG073114 | Brd2 |
| Btg3 associated nuclear protein | BG067472 | Banp |
| calcium channel, voltage-dependent, beta 3 subunit | BG072959 | Cacnb3 |
| calmodulin 2 | AW537940 | Calm2 |
| casein kinase 1, epsilon | BG080793 | Csnk1e |
| catenin src | C77281 | Catns |
| cathepsin 8 | BG064382 | Cts8 |
| CCAAT/enhancer binding protein alpha (C/EBP), related sequence 1 | BG064857 | Cebpa-rs1 |
| CCR4-NOT transcription complex, subunit 2 | BG066658 | Cnot2 |
| CD84 antigen | BG069587 | Cd84 |
| CD9 antigen | BG087410 | Cd9 |
| CDC-like kinase | BG065099 | Clk |
| CDP-diacylglycerol--inositol 3-phosphatidyltransferase (phosphatidylinositol synthase) | BG065447 | Cdipt |
| cell division cycle 25 homolog C (S. cerevisiae) | BG079263 | Cdc25c |
| cell division cycle 6 homolog (S. cerevisiae) | BG077012 | Cdc6 |
| chorionic somatomammotropin hormone 1 | BG063848 | Csh1 |
| chromatin assembly factor 1, subunit A (p150) | BG070452 | Chaf1a |
| chromobox homolog 5 (Drosophila HP1a) | BG078574 | Cbx5 |
| coatomer protein complex, subunit beta 2 (beta prime) | BG065398 | Copb2 |
| coproporphyrinogen oxidase | BG080324 | Cpox |
| coronin, actin binding protein 1B | BG064358 | Coro1b |
| cyclin D2 | AW547625 | Ccnd2 |
| cyclin G | BG065754 | Ccng1 |
| cysteine and histidine-rich domain (CHORD)-containing, zinc-binding protein 1 | BG066642 | Chordc1 |
| cytochrome b-245, beta polypeptide | BG081063 | Cybb |
| cytochrome P450, 17 | BG064307 | Cyp17a1 |
| cytokine inducible SH2-containing protein 3 | BG076991 | Socs3 |
| cytosolic aminopeptidase P | BG078715 | Xpnpep1 |
| cytotoxic granule-associated RNA binding protein 1 | BG076908 | Tia1 |
| DEAD/H (Asp-Glu-Ala-Asp/His) box polypeptide 15 (RNA helicase A) | BG065486 | Dhx15 |
| DEAD/H (Asp-Glu-Ala-Asp/His) box polypeptide 19 | BG066118 | 2810457M08Rik |
| dihydrofolate reductase | BG078582 | Dhfr |
| DNA segment, Chr 1, ERATO Doi 185, expressed | BG066240 | D1Ertd185e |
| DNA segment, Chr 1, ERATO Doi 309, expressed | C80376 | D1Ertd309e |
| DNA segment, Chr 10, ERATO Doi 447, expressed | BG067467 | D10Ertd447e |
| DNA segment, Chr 10, ERATO Doi 709, expressed | BG069224 | D10Ertd709e |
| DNA segment, Chr 10, Wayne State University 102, expressed | BG069391 | D10Wsu102e |
| DNA segment, Chr 10, Wayne State University 93, expressed | BG063108 | D10Wsu93e |
| DNA segment, Chr 11, ERATO Doi 752, expressed | BG065401 | 1810022J16Rik |
| DNA segment, Chr 11, ERATO Doi 768, expressed | BG069684 | Mettl2 |
| DNA segment, Chr 11, Wayne State University 68, expressed | BG064501 | D11Wsu68e |
| DNA segment, Chr 14, ERATO Doi 170, expressed | BG066185 | 1810063B07Rik |
| DNA segment, Chr 15, ERATO Doi 180, expressed | BG066215 | D15Ertd180e |
| DNA segment, Chr 15, ERATO Doi 320, expressed | BG066842 | D15Ertd320e |
| DNA segment, Chr 16, ERATO Doi 88, expressed | C77945 | D16Ertd88e |
| DNA segment, Chr 17, ERATO Doi 657, expressed | BG070247 | D17Ertd657e |
| DNA segment, Chr 19, Wayne State University 162, expressed | BG063696 | D19Wsu162e |
| DNA segment, Chr 2, ERATO Doi 337, expressed | BG066920 | D2Ertd337e |
| DNA segment, Chr 2, ERATO Doi 92, expressed | BG065844 | D2Ertd92e |
| DNA segment, Chr 4, ERATO Doi 103, expressed | BG065883 | D4Ertd103e |
| DNA segment, Chr 4, ERATO Doi 111, expressed | BG066050 | D4Ertd111e |
| DNA segment, Chr 4, ERATO Doi 429, expressed | BG067358 | AV028368 |
| DNA segment, Chr 4, ERATO Doi 58, expressed | BG066000 | D4Ertd58e |
| DNA segment, Chr 5, ERATO Doi 77, expressed | BG065860 | D5Ertd77e |
| DNA segment, Chr 6, ERATO Doi 456, expressed | BG067549 | Igk-V8 |
| DNA segment, Chr 6, ERATO Doi 87, expressed | C77948 | D6Ertd87e |
| DNA segment, Chr 7, ERATO Doi 187, expressed | C78607 | Nalp6 |
| DNA segment, Chr 7, ERATO Doi 193, expressed | BG066262 | D7Ertd193e |
| DNA segment, Chr 7, ERATO Doi 443, expressed | BG067408 | D7Ertd443e |
| DNA segment, Chr 7, ERATO Doi 715, expressed | BG069287 | D7Ertd715e |
| DNA segment, Chr 7, ERATO Doi 764, expressed | BG069589 | Kcnk6 |
| DNA segment, Chr 8, ERATO Doi 107, expressed | BG079141 | D8Ertd107e |
| DNA segment, Chr X, ERATO Doi 223, expressed | BG066388 | DXErtd223e |
| DnaJ (Hsp40) homolog, subfamily B, member 6 | BG076720 | Dnajb6 |
| dynein, cytoplasmic, heavy chain 1 | BG072963 | Dnchc1 |
| EH-domain containing 1 | BG063097 | Ehd1 |
| endoplasmic reticulum (ER) to nucleus signalling 1 | BG080244 | Ern1 |
| endoplasmic reticulum resident protein 44kDa | BG077695 | Txndc4 |
| epithelial protein lost in neoplasm | BG079450 | D15Ertd366e |
| espin | BG068280 | Espn |
| ethanol induced 6 | BG077332 | LOC366603 |
| eukaryotic translation initiation factor 2, subunit 2 (beta, 38kDa) | BG063608 | Eif2s2 |
| eukaryotic translation initiation factor 2A | BG085504 | Eif2s1 |
| eukaryotic translation initiation factor 3 | BG076674 | Eif3s10 |
| expressed sequence AA408298 | BG076670 | 4921515A04Rik |
| expressed sequence AA408683 | BG063272 | 5730472N09Rik |
| expressed sequence AA408877 | BG081577 | D1Ucla4 |
| expressed sequence AA410048 | BG076471 | 4432406C08Rik |
| expressed sequence AA410158 | BG067405 | Gtl6 |
| expressed sequence AA589532 | BG062977 | AA589532 |
| expressed sequence AI046671 | BG065399 | 2900010J23Rik |
| expressed sequence AI116001 | BG067550 | D8Ertd457e |
| expressed sequence AI303526 | BG081027 | AI303526 |
| expressed sequence AI327276 | BG076924 | AI327276 |
| expressed sequence AI385631 | BG067444 | Trim46 |
| expressed sequence AI447318 | BG067401 | 1700009P03Rik |
| expressed sequence AI450313 | BG077241 | AI450313 |
| expressed sequence AI452301 | BI076508 | AI452301 |
| expressed sequence AI462493 | BG076190 | AI462493 |
| expressed sequence AI467481 | BG082688 | AI467481 |
| expressed sequence AI481284 | BG067482 | L3mbtl3 |
| expressed sequence AI481289 | BG073776 | Il1rl2 |
| expressed sequence AI643885 | C76157 | AI643885 |
| expressed sequence AI649097 | BG073155 | 9030625G08Rik |
| expressed sequence AI787263 | BG065385 | Slc31a1 |
| expressed sequence AI987944 | BG087446 | AI987944 |
| expressed sequence AL022637 | BG065372 | A930034L06Rik |
| expressed sequence AU015105 | BG083097 | Stx1bl |
| expressed sequence AU015154 | BG070198 |  |
| expressed sequence AU018493 | BG082264 | Ccnl1 |
| expressed sequence AU018638 | BG067350 | BC018601 |
| expressed sequence AU018728 | BG069308 | 2900026H06Rik |
| expressed sequence AU019351 | BG069378 | Mcrs1 |
| expressed sequence AU020745 | BG069797 | AU020745 |
| expressed sequence AU021725 | BG068026 | AU021725 |
| expressed sequence AU022252 | BG068186 | AU022252 |
| expressed sequence AU022554 | BG068311 | AU022554 |
| expressed sequence AU042020 | BG077439 | Plxnb1 |
| expressed sequence AU042359 | BG065000 | Abr |
| expressed sequence AV002070 | BG069617 | Kremen |
| expressed sequence AW047581 | BG077928 | C130032J12Rik |
| expressed sequence AW060987 | BG065289 | Ablim1 |
| expressed sequence AW146002 | BG065404 | 1700055P21Rik |
| expressed sequence AW536573 | BG078302 | Lars |
| expressed sequence AW536594 | BG065011 | Nmt1 |
| expressed sequence C76554 | BG065701 | C76554 |
| expressed sequence C77591 | BG065977 | C77591 |
| expressed sequence C78409 | BG066196 | D630010I07 |
| expressed sequence C78444 | BG066204 | C78444 |
| expressed sequence C78505 | BG066222 | C78505 |
| expressed sequence C78859 | C78859 | C78859 |
| expressed sequence C78880 | BG066338 | C78880 |
| expressed sequence C78997 | BG066380 | Gtl6 |
| expressed sequence C79491 | BG066607 | C79491 |
| expressed sequence C79563 | BG066628 | C79563 |
| expressed sequence C79601 | BG066640 | C79601 |
| expressed sequence C79657 | BG066659 | C79657 |
| expressed sequence C79672 | BG066663 | A430005L14Rik |
| expressed sequence C80161 | BG066733 | C80161 |
| expressed sequence C80406 | BG066799 | C80406 |
| expressed sequence C80425 | BG066804 | C80425 |
| expressed sequence C80446 | BG066813 | C80446 |
| expressed sequence C80571 | BG066835 | C80571 |
| expressed sequence C80758 | BG064208 | C80758 |
| expressed sequence C80914 | BG066905 | C80914 |
| expressed sequence C80918 | BG066907 | C80918 |
| expressed sequence C80993 | BG067016 | C80993 |
| expressed sequence C85340 | C85340 | 1110059P08Rik |
| expressed sequence C86544 | BG067503 | C86544 |
| expressed sequence C86753 | BG067565 | C86753 |
| expressed sequence C86896 | BG067607 | C86896 |
| expressed sequence C86933 | BG080756 | C86933 |
| expressed sequence C87011 | BG067811 | C87011 |
| expressed sequence C87222 | BG086892 | Arhgdia |
| expressed sequence C87251 | BG067720 | BC036333 |
| expressed sequence C87398 | BG067759 | C87251 |
| expressed sequence C87482 | BG067265 | C87482 |
| farnesyl diphosphate farnesyl transferase 1 | BG069211 | Fdft1 |
| fibroblast growth factor 10 | BG066324 | Fgf10 |
| FK506 binding protein 4 (59 kDa) | BG064128 | Fkbp4 |
| flightless I homolog (Drosophila) | C79694 | Flii |
| forkhead box J2 | BG075205 | Foxj2 |
| G two S phase expressed protein 1 | BG073090 | Gtse1 |
| gap junction membrane channel protein beta 5 | BG077559 | Gjb5 |
| gene rich cluster, C8 gene | C81126 | Cdca3 |
| gene trap locus 1-13 | BG077939 | Nup160 |
| general transcription factor II I | BG074917 | Gtf2i |
| general transcription factor III C 1 | AU015927 | Gtf3c1 |
| glucose phosphate isomerase 1 complex | BG080751 | Gpi1 |
| glyceronephosphate O-acyltransferase | BG069252 | Gnpat |
| growth arrest and DNA-damage-inducible 45 gamma | BG067419 | Gadd45g |
| growth differentiation factor 9 | C86021 | Gdf9 |
| H19 fetal liver mRNA | BG087529 | h19 |
| H2A histone family, member Z | BG065111 | H2afz |
| H2A histone family, member Z | BG065110 | H2afz |
| H2A histone family, member Z | BG076995 | H2afz |
| heat shock 70kD protein 8 | BG087043 | Hspa8 |
| hematological and neurological expressed sequence 1 | BG066697 | Hn1 |
| heme oxygenase (decycling) 1 | BG077732 | Hmox1 |
| hemoglobin alpha, adult chain 1 | BG073467 | Hba-a1 |
| hemoglobin Y, beta-like embryonic chain | BG073045 | Hbb-y |
| hepatoma-derived growth factor | BG066728 | Hdgf |
| heterogeneous nuclear ribonucleoprotein A2/B1 | BG064117 | Hnrpa2b1 |
| histidine triad nucleotide binding protein | BG077758 | Hint1 |
| Hoxa1 regulated gene | BG074406 | AL022832 |
| hydroxysteroid 17-beta dehydrogenase 4 | BG072994 | Hsd17b4 |
| hypothetical protein, clone 2-24 | BG063313 | 2410016F01Rik |
| immediate early response, erythropoietin 1 | BG080722 | Copeb |
| inhibitor of growth family, member 1 | BG063128 | Ing1 |
| inner membrane protein, mitochondrial | AW555640 | Immt |
| inositol 1,4,5-triphosphate receptor 5 | BG067589 | Itpr5 |
| interferon alpha responsive gene, 15 kDa | BG078215 | Ifrg15 |
| interferon regulatory factor 1 | BG067127 | Irf1 |
| Janus kinase 2 | BG068848 | Jak2 |
| jerky | BG085919 | Jrk |
| karyopherin (importin) beta 3 | BG078314 | Kpnb3 |
| kelch-like 2, Mayven (Drosophila) | BG069796 | Klhl2 |
| kinesin family member 5B | BG063047 | Kif5b |
| lactotransferrin | BG085146 | Ltf |
| lectin, galactose binding, soluble 1 | BG078028 | Lgals1 |
| lectin, galactose binding, soluble 3 | BG064176 | lgals3 |
| LIM domains containing 1 | BG069785 | Limd1 |
| lipoprotein lipase | BG076860 | Kctd5 |
| Lutheran blood group (Auberger b antigen included) | AW553617 | Lu |
| M.musculus mRNA for glutamyl-tRNA synthetase | BG064194 |  |
| makorin, ring finger protein, 3 | BG076438 | Sfn |
| mammary tumor integration site 6 | BG078803 | Eif3s6 |
| mannosidase 1, beta | BG080131 | Man1b |
| mannosidase 2, alpha B1 | BG066363 | Man2b1 |
| methylenetetrahydrofolate dehydrogenase (NADP+ dependent), methenyltetrahydrofolate cyclohydrolase, formyltetrahydrofolate synthase | BG087719 | Mthfd1 |
| methyltransferase Cyt19 | AU020528 | 2310045H08Rik |
| mini chromosome maintenance deficient (S. cerevisiae) | BG065055 | Mcm3 |
| minichromosome maintenance deficient (S. cerevisiae) 3-associated protein | C79187 | Mcm3ap |
| mitochondrial ribosomal protein L4 | BG086523 | Mrpl4 |
| mitochondrial ribosomal protein S15 | BG071485 | Mrps15 |
| mitogen activated protein kinase 13 | BG083840 | Mapk13 |
| Mpv17 transgene, kidney disease mutant | BG066244 | Mpv17 |
| Mus musculus evectin-2 (Evt2) mRNA, complete cds | BG075360 | Plekhb2 |
| Mus musculus sialic acid synthase (Sas), mRNA | BQ550102 | Nans |
| Mus musculus, clone IMAGE:3586067, mRNA, partial cds | BG072997 | B230379M23Rik |
| Mus musculus, clone IMAGE:3963643, mRNA, partial cds | BG070884 | BC037006 |
| Mus musculus, clone IMAGE:3995747, mRNA, partial cds | BG063804 | Slc39a14 |
| Mus musculus, clone IMAGE:4236601, mRNA | BG067445 | Kpna6 |
| Mus musculus, clone IMAGE:5342828, mRNA, partial cds | BQ551086 | E130317O14Rik |
| Mus musculus, clone IMAGE:5375863, mRNA, partial cds | BQ551707 | 9630050M13Rik |
| Mus musculus, clone MGC:28734 IMAGE:4460615, mRNA, complete cds | BG065707 | Tbl3 |
| Mus musculus, clone MGC:36285 IMAGE:4163356, mRNA, complete cds | BG082399 | Mocs1 |
| Mus musculus, clone MGC:36911 IMAGE:4945500, mRNA, complete cds | BG081030 | Plekha4 |
| Mus musculus, clone MGC:7054 IMAGE:3156506, mRNA, complete cds | BG068349 | 6430706D22Rik |
| Mus musculus, clone MGC:7480 IMAGE:3490700, mRNA, complete cds | BG069660 | Cugbp1 |
| Mus musculus, Similar to dysferlin, clone IMAGE:5324940, mRNA, partial cds | BG064461 | Dysf |
| Mus musculus, similar to H2A histone family, member O, clone MGC:36202 IMAGE:5055276, mRNA, complete cds | BG063040 | Hist1h2ao |
| Mus musculus, Similar to hypothetical gene LOC133157, clone IMAGE:3991705, mRNA, partial cds | BG063780 |  |
| Mus musculus, Similar to hypothetical protein C321D2.4, clone MGC:38008 IMAGE:5149957, mRNA, complete cds | BG063423 | Chtf18 |
| Mus musculus, Similar to hypothetical protein FLJ10008, clone MGC:38228 IMAGE:5323598, mRNA, complete cds | BG067060 | BC002230 |
| Mus musculus, Similar to hypothetical protein FLJ10044, clone MGC:19129 IMAGE:4212305, mRNA, complete cds | BG082740 | Lims2 |
| Mus musculus, Similar to hypothetical protein FLJ10359, clone MGC:30806 IMAGE:3992793, mRNA, complete cds | BG069240 | BC019693 |
| Mus musculus, Similar to hypothetical protein FLJ22479, clone IMAGE:4487274, mRNA, partial cds | BG073791 |  |
| Mus musculus, Similar to hypothetical protein FLJ22693, clone IMAGE:5059780, mRNA, partial cds | BG081062 | Zc3hdc1 |
| Mus musculus, Similar to KIAA1404 protein, clone IMAGE:5252426, mRNA, partial cds | BG080292 | AI481105 |
| Mus musculus, Similar to phosphoinositol 3-phosphate-binding protein-2, clone MGC:11882 IMAGE:3598156, mRNA, complete cds | BG067450 |  |
| Mus musculus, Similar to solute carrier family 22 (organic anion transporter), member 7, clone MGC:18877 IMAGE:4236556, mRNA, complete cds | BG078261 | Slc22a7 |
| Mus musculus, Similar to solute carrier family 6 (neurotransmitter transporter, GABA), member 13, clone MGC:28956 IMAGE:4240641, mRNA, complete cds | BG075861 | Slc6a13 |
| Mus musculus, Similar to step II splicing factor SLU7, clone IMAGE:3602931, mRNA, partial cds | BG077767 | D11Ertd730e |
| Mus musculus, Similar to ubiquitin specific protease 1, clone MGC:25528 IMAGE:3585191, mRNA, complete cds | BG066359 | Usp1 |
| Mus musculus, Similar to ubiquitin specific protease 3, clone MGC:28886 IMAGE:4911201, mRNA, complete cds | BG069818 | Usp3 |
| Mus musculus, Similar to xylosylprotein beta1,4-galactosyltransferase, polypeptide 7 (galactosyltransferase I), clone MGC:28643 IMAGE:4224150, mRNA, complete cds | BG064673 | B4galt7 |
| Mus musculus, translocase of inner mitochondrial membrane 10 homolog (yeast), clone MGC:35982 IMAGE:4976059, mRNA, complete cds | BQ550445 | Timm10 |
| myocyte enhancer factor 2D | BG080755 | 2210408E11Rik |
| natrium-phosphate cotransporter IIa C-terminal-associated protein 2 | BG069369 | Pdzk2 |
| N-deacetylase/N-sulfotransferase (heparan glucosaminyl) 2 | BG085858 | Ndst2 |
| necdin | BG079634 | Ndn |
| neuraminidase 1 | BG063881 | Neu1 |
| neuroblastoma ras oncogene | BG064144 | nras |
| neuroepithelial cell transforming gene 1 | BG083317 | Net1 |
| nicastrin | BG063777 | ncstn |
| NIMA (never in mitosis gene a)-related expressed kinase 7 | BG069659 | Nek7 |
| NMDA receptor-regulated gene 1 | BG078091 | Narg1 |
| nuclear protein 1 | BG084947 | Nupr1 |
| nuclear protein 95 | BG065028 | Uhrf1 |
| nuclear RNA export factor 1 homolog (S. cerevisiae) | BG077817 | Nxf1 |
| nuclear, factor, erythroid derived 2, like 2 | BG085841 | Nfe2l2 |
| nucleolin | BG064932 | Ncl |
| oviductal glycoprotein 1, 120kD | BG080500 | Ovgp1 |
| p53 apoptosis effector related to Pmp22 | BG065306 | Perp |
| peroxisomal biogenesis factor 11a | BG064512 | Ap3s2 |
| phosphatidylinositol 3-kinase catalytic delta polypeptide | BG077230 | Pik3cd |
| phospholipase c neighboring | BG080489 | Ppp1r14b |
| phosphotyrosyl phosphatase activator | AW556496 | ptpa |
| PIN2/TRF1-interacting protein | BG081900 | Pinx1 |
| podocalyxin-like | BG086980 | Ccnd2 |
| procollagen, type IV, alpha 1 | BG085352 | Col4a1 |
| procollagen, type V, alpha 2 | BG076180 | Col5a2 |
| programmed cell death 5 | BG076705 | Pdcd5 |
| prolactin-like protein A | AA408595 | Prlpa |
| proliferating cell nuclear antigen | BG064598 | Pcna |
| proliferation related acidic leucine rich protein PAL31 | BG065023 |  |
| prosaposin | BG088310 | Psap |
| protein phosphatase 4, catalytic subunit | BG071232 | Ppp4c |
| puromycin-sensitive aminopeptidase | BG065063 | Psa |
| putative homeodomain transcription factor | BG070102 | Phtf1 |
| RAB geranylgeranyl transferase, b subunit | BG076913 | Rabggtb |
| RAB14, member RAS oncogene family | BG064879 | Rab14 |
| RAB3D, member RAS oncogene family | BG069254 | MGC18837 |
| reduced expression 3 | BG064920 | Rex3 |
| regulatory factor X-associated ankyrin-containing protein | BG077353 | Rfxank |
| ribosomal protein L18 | AA409347 | rpl18 |
| ribosomal protein L26 | BG078672 | rpl26 |
| ribosomal protein L27a | BG079639 | Rpl27a |
| ribosomal protein L5 | BG065194 | Rpl5 |
| ribosomal protein L7 | BG063883 | Rpl7 |
| ribosomal protein L8 | BG077480 | Rpl8 |
| ribosomal protein S18 | BG076809 | Rps18 |
| ribosomal protein S6 kinase polypeptide 1 | BG079900 | Rps6ka1 |
| ribosome binding protein 1 | BG077002 | Rrbp1 |
| roundabout homolog 1 (Drosophila) | BG078518 | Robo1 |
| runt related transcription factor 2 | BG067185 | Runx2 |
| S100 calcium binding protein A13 | AW542372 | S100a13 |
| SAR1a gene homolog (S. cerevisiae) | BG077753 | Sara1 |
| selectin, endothelial cell, ligand | BG080352 | Glg1 |
| sema domain, immunoglobulin domain (Ig), short basic domain, secreted, (semaphorin) 3E | BG078687 | Sema3e |
| serine hydroxymethyl transferase 1 (soluble) | BG078187 | Shmt1 |
| serine/threonine kinase 25 (yeast) | BG069228 | Stk25 |
| SH3 domain protein 2A | BG087707 | Sh2d2a |
| sideroflexin 1 | BG078316 | Sfxn1 |
| small nuclear ribonucleoprotein E | BG085367 | Snrpe |
| smoothelin | BG066516 | Smtn |
| soc-2 (suppressor of clear) homolog (C. elegans) | BG087119 | Shoc2 |
| solute carrier family 15 (H+/peptide transporter), member 2 | BG079545 | Slc15a2 |
| solute carrier family 25 (mitochondrial carrier; adenine nucleotide translocator), member 13 | BG083930 | Slc25a13 |
| solute carrier family 25 (mitochondrial carrier; oxoglutarate carrier), member 11 | BG083931 | Slc25a11 |
| solute carrier family 25 (mitochondrial deoxynucleotide carrier), member 19 | AW538113 | Slc25a19 |
| solute carrier family 28 (sodium-coupled nucleoside transporter), member 2 | BG077361 | Slc28A2 |
| solute carrier family 29 (nucleoside transporters), member 2 | BG065304 | Slc29a2 |
| solute carrier family 9 (sodium/hydrogen exchanger), isoform 3 regulator 1 | BG066200 | Slc9a3r1 |
| spectrin SH3 domain binding protein 1 | BG065421 | Abi1 |
| splicing factor, arginine/serine-rich 3 (SRp20) | BG064952 | Sfrs3 |
| stearoyl-Coenzyme A desaturase 2 | BG066641 | Scd2 |
| sterol O-acyltransferase 1 | BG087432 | Soat1 |
| succinate-Coenzyme A ligase, ADP-forming, beta subunit | BG069442 | Sucla2 |
| sudD, suppressor of bimD6 homolog (Aspergillus nidulans) | BG066885 | Riok3 |
| suppression of tumorigenicity 7 | BG068896 | St7 |
| suppressor of initiator codon mutations, related sequence 1 (S. cerevisiae) | BG086987 | Sui1-rs1 |
| SWI/SNF related, matrix associated, actin dependent regulator of chromatin, subfamily d, member 1 | BG065201 | Smarcd1 |
| synaptosomal-associated protein, 23kD | BG083050 | Snap23 |
| TAF12 RNA polymerase II, TATA box binding protein (TBP)-associated factor, 20 kDa | BG075593 | Taf12 |
| testis expressed gene 189 | BG064029 | Morf4l1 |
| tetratricopeptide repeat domain | BG063181 | Ttc3 |
| thioredoxin-like (32kD) | BG071869 | Txnl |
| thymosin, beta 10 | BG063081 | Tmsb10 |
| toll interacting protein | C79165 | Tollip |
| toll-interleukin 1 receptor (TIR) domain-containing adaptor protein | BG065379 | Tirap |
| tousled-like kinase 2 (Arabidopsis) | BG071806 | Tlk2 |
| TPR-containing, SH2-binding phosphoprotein | BG076810 | Sh2bp1 |
| trans-acting transcription factor 1 | BG077567 | D030041N15Rik |
| transforming growth factor beta 1 induced transcript 4 | BG081592 | Tgfb1i4 |
| translocase of inner mitochondrial membrane 8 homolog b (yeast) | BG080049 | Timm8b |
| tropomyosin 2, beta | BG087093 | Tpm2 |
| tubulin, beta 3 | BG080671 | Tubb3 |
| tubulin, beta 5 | BG087420 | Tubb5 |
| tyrosine 3-monooxygenase/tryptophan 5-monooxygenase activation protein, beta polypeptide | BG085811 | Ywhab |
| tyrosine 3-monooxygenase/tryptophan 5-monooxygenase activation protein, zeta polypeptide | BG065186 | Pdap1 |
| ubiquinol-cytochrome c reductase core protein 1 | BG069853 | Uqcrc1 |
| ubiquitin protein ligase E3A | AW537395 | Ube3a |
| ubiquitin specific protease 2 | BG070961 | Usp2 |
| ubiquitin specific protease 21 | BG065003 | Usp21 |
| ubiquitin specific protease 25 | BG075872 | Usp25 |
| ubiquitin-conjugating enzyme E2 variant 2 | BG076826 | Ube2v2 |
| ubiquitin-conjugating enzyme E2N | C87709 | Ube2n |
| UDP-Gal:betaGlcNAc beta 1,3-galactosyltransferase, polypeptide 4 | BG077355 | B3galt4 |
| UDP-galactose translocator 2 | BG064404 | Ugalt2 |
| unc5 homolog (C. elegans) 3 | BG070534 | Unc5c |
| upstream transcription factor 2 | BG078718 | 1810073K19Rik |
| valosin containing protein | AW552886 | Vcp |
| Y box protein 3 | BG079767 | Csda |
| yolk sac gene 2 | BG064209 | Siae |
| zinc finger RNA binding protein | C80485 | Zfr |
