# Supplementary material for: Gonadal Transcriptome Alterations in Response to Dietary Energy Intake: Sensing the Reproductive Environment
Source: PLoS One. 2009 Jan 7;4(1):e4146. doi: 10.1371/journal.pone.0004146 (PMC2607546; doi:10.1371/journal.pone.0004146)
Supplement: Table S2 — PAGE pathway abbreviations: Molecular Signatures Database (www.broad.mit.edu/gsea/msigdb/genesets.jsp) (0.41 MB DOC) [file pone.0004146.s010.doc]

**Table S2: PAGE pathway abbreviations: Molecular Signatures Database (www.broad.mit.edu/gsea/msigdb/genesets.jsp)**

**Figure 9: PAGE pathway abbreviations.**

| **Pathway abbreviation** | **Pathway name** |
| --- | --- |
| ANTI_CD44 | ANTI-CD44_UP |
| Butanoate | MAP00650_Butanoate_metabolism |
| Dictyostel - ST | Dictyostelium_discoideum_cAMP_Chemotaxis_Pathway |
| Fattyacidbsy | MAP00062_Fatty_acid_biosynthesis_path_2 |
| Glycans | MAP00512_O_Glycans_biosynthesis |
| Inositol | MAP00562_Inositol_phosphate_metabolism |
| KET | Genes involved in KETONE processing |
| PIP3 | SIG_PIP3SIGINCARDIACMYOCTES |
| Propanoate | MAP00640_Propanoate_metabolism |
| SA_PTEN | SA_PTEN_PATHWAY |
| ST_Diff | ST_Differentiation_Pathway_in_PC12_Cells |
| Starch | MAP00500_Starch_and_sucrose_metabolism |
| Valine | MAP00280_Valine_leucine_and_isoleucine_degradation |
| Cd40 | cd40Pathway |
| cdmac | cdmacPathway |
| ctl | ctlPathway |
| etc | etcPathway |
| ets | etsPathway |
| fosb | fosbPathway |
| malate | malatePathway |
| malatex | malatexPathway |
| nfkb | nfkbPathway |
| notch | notchPathway |
| pkc | pkcPathway |
| plcd | plcdPathway |
| ps1 | ps1Pathway |
| ptdins | ptdinsPathway |
| rna | rnaPathway |
| shh_lisa | shh_lisa |
| ketone | ketonebodiesPathway |
| lair | lairPathway |
| GNFFEMALE | GNF_FEMALE_GENES |
| Complement | Complement_Activation_Classical |
| SAG2ANDM | SA_G2_AND_M_PHASES |
| argininec | argininecPathway |
| tnfandfas | tnf_and_fas_network |
| gs | gsPathway |
| INSULIN2F_DOWN | INSULIN_2F_DOWN |
| DAG1 | SA_DAG1 |
| Glutathione | MAP00480_Glutathione_metabolism |
| Erbb4 | erbb4Pathway |
| Igf1mtor | igf1mtorPathway |
| IL17 | il17Pathway |
| Slrp | slrpPathway |
| hbx | hbxPathway |
| SIG_CHEMOTAX | SIG_CHEMOTAXIS |
| AlaAsp | MAP00252_Alanine_and_aspartate_metabolism |
| CR_TRANS | CR_TRANSCRIPTION_FACTORS |
| CitrateTCA | MAP00020_Citrate_cycle_TCA_cycle |
| Cysteine | MAP00272_Cysteine_metabolism |
| G13 | G13_Signaling_Pathway |
| GLUCOSE_UP | GLUCOSE_UP |
| Globoside | MAP00603_Globoside_metabolism |
| Krebs-TCA | Krebs-TCA_Cycle |
| Nitrogen | MAP00910_Nitrogen_metabolism |
| PGC | PGC |
| Rho-GTPase | SIG_Regulation_of_the_actin_cytoskeleton_by_Rho_GTPases |
| Steroid | MAP00140_C21_Steroid_hormone_metabolism |
| TCA | TCA |
| TRKA | SA_TRKA_RECEPTOR |
| Wnt_Ca2 | ST_Wnt_Ca2_cyclic_GMP_Pathway |
| ace2 | ace2Pathway |
| app | appPathway |
| cacamPathway | cell_adh - cell_adhesion_molecule_activity |
| cell_adhact | cell_adhesion_receptor_activity |
| cftr | cftrPathway |
| chrebp | chrebpPathway |
| eph4A | ephA4Pathway |
| gcr | gcrPathway |
| hsp27 | hsp27Pathway |
| inflam | inflamPathway |
| lechtin | lechtinPathway |
| rho | rhoPathway |
| salmonella | salmonellaPathway |
| set | setPathway |
| sodd | soddPathway |
| c_c_c | cell_cycle_checkpoint |
| eif4 | eif4Pathway |
| extrinsic | extrinsicPathway |
| intrinsic | intrinsicPathway |
| pparg | ppargPathway |
| Stress | stressPathway |
| Bile_acid | MAP00120_Bile_acid_biosynthesis |
| IMMUNE | CR_IMMUNE_FUNCTION |
| MAPK_Cas | MAPK_Cascade |
| ROS | ROS |
| atm | atmPathway |

**Figure 10: PAGE pathway abbreviations.**

| **Pathway abbreviation** | **Pathway name** |
| --- | --- |
| Benzoate | MAP00632_Benzoate_degradation |
| CD44_UP | ANTI_CD44_UP |
| FETAL_LIVER | FETAL_LIVER_HS_ENRICHED_TF_JP |
| Fattyacidbsy | MAP00062_Fatty_acid_biosynthesis_path_2 |
| GLUCO | GLUCO |
| GLUT_DOWN | GLUT_DOWN |
| GLYCOGEN | GLYCOGEN |
| KET | Genes involved in KETONE processing |
| PROLIF_GENE | PROLIF_GENES |
| Propanoate | MAP00640_Propanoate_metabolism |
| MMP | SA_MMP_CYTOKINE_CONNECTION |
| Valine | MAP00280_Valine_leucine_and_isoleucine_degradation |
| electrnspract | electron_transporter_activity |
| cd40 | cd40Pathway |
| cell_prolif | cell_proliferation |
| cellcyclearr | cell_cycle_arrest |
| eif2 | eif2Pathway |
| etc | etcPathway |
| ets | etsPathway |
| inflam | inflamPathway |
| ldl | ldlPathway |
| nthi | nthiPathway |
| XINACT | XINACT_MERGED |
| cell_motil | cell_motility |
| classic | classicPathway |
| comp | compPathway |
| edg1 | edg1Pathway |
| p27 | p27Pathway |
| Tert | tertPathway |
| CYCLIN | SA_REG_CASCADE_OF_CYCLIN_EXPR |
| GPI7T | ST_G_Protein_Independent_7_Transmembrane |
| Skp2e2f | skp2e2fPathway |
| Steroid | MAP00140_C21_Steroid_hormone_metabolism |
| GPI7TRS | ST_G_Protein_Independent_7_Transmembrane_Receptor_Signaling |
| G_alpha_i | ST_G_alpha_i_Pathway |
| MAP_kinase | MAP_kinase_activity |
| PROGRAM | SA_PROGRAMMED_CELL_DEATH |
| CR_TSPT | CR_TRANSPORT |
| Complement | Complement_Activation_Classical |
| Glycolysis | glycolysisPathway |
| Igf12mtor | igf1mtorPathway |
| cacam | cacamPathway |
| MMP | SA_MMP_CYTOKINE_CONNECTION |
| FAS | SA_FAS_SIGNALLING |
| GLUCOSE_UP | GLUCOSE_UP |
| glycans | MAP00512_O_Glycans_biosynthesis |
| Sterol | MAP00100_Sterol_biosynthesis |
| Urea_cycle | MAP00220_Urea_cycle_and_metabolism_of_amino_groups |
| Wnt_Ca2 | ST_Wnt_Ca2_cyclic_GMP_Pathway |
| cpt | cptPathway |
| hbx | hbxPathway |
| hsp27 | hsp27Pathway |
| il10 | il10Pathway |
| sodd | soddPathway |
| intrinsic | intrinsicPathway |
| 5739 | GO_0005739 |
| Bile_acid | MAP00120_Bile_acid_biosynthesis |
| DOWNHOXA9 | DOWNREG_BY_HOXA9 |
| ETChain | Electron_Transport_Chain |
| HOXA9_DOWN | HOXA9_DOWN |
| ROS | ROS |
| VOXPHOS | VOXPHOS |
| malate | malatePathway |
| mta3 | mta3Pathway |
| plk3 | plk3Pathway |
| pparg | ppargPathway |
| ps1 | ps1Pathway |
| Sppa | sppaPathway |
| Wnt_signal | Wnt_Signaling |
| ach | achPathway |
| aif | aifPathway |
| cdc25 | cdc25Pathway |

**Figure 11: PAGE pathway abbreviations.**

| **Pathway abbreviation** | **Pathway name** |
| --- | --- |
| Fattyacidbsy | MAP00062_Fatty_acid_biosynthesis_path_2 |
| Glyoxylate | MAP00630_Glyoxylate_and_dicarboxylate_metabolism |
| KET | Genes involved in KETONE processing |
| g2 | g2Pathway |
| Bile_acid | MAP00120_Bile_acid_biosynthesis |
| atm | atmPathway |
| Valine | MAP00280_Valine_leucine_and_isoleucine_degradation |
| Methionine | MAP00271_Methionine_metabolism |
| One_carbon | MAP00670_One_carbon_pool_by_folate |
| glycolysis | glycolysisPathway |
| ldl | ldlPathway |
| nfkb | nfkbPathway |
| pkc | pkcPathway |
| ketone | ketonebodiesPathway |
| reck | reckPathway |
| rela | relaPathway |
| myosin | myosinPathway |
| tnfr2 | tnfr2Pathway |
| tob1 | tob1Pathway |
| gs | gsPathway |
| FAS | SA_FAS_SIGNALLING |
| Shh_lisa | Shh_lisa |
| gaba | gabaPathway |
| egfr_smrte | egfr_smrtePathway |
| carm1 | carm1Pathway |
| muscle_myosin | muscle_myosin |
| FRASOR_DOWN | FRASOR_ER_DOWN |
| il17 | il17Pathway |
| ANDROGEN | ANDROGEN_GENES_FROM_NETAFFX |
| ami | amiPathway |
| cpt | cptPathway |
| csk | cskPathway |
| etc | etcPathway |
| gata3 | gata3Pathway |
| BCR_SIG | SIG_BCR_Signaling_Pathway |
| Methane | MAP00680_Methane_metabolism |
| CO2-fixation | MAP00720_Reductive_carboxylate_cycle_CO2_fixation |
| AR_MOUSE | AR_MOUSE |
| SIG_CHEMOTAX | SIG_CHEMOTAXIS |
| CitrateTCA | MAP00020_Citrate_cycle_TCA_cycle |
| Cysteine | MAP00272_Cysteine_metabolism |
| Dictyostel | ST_Dictyostelium_discoideum_cAMP_Chemotaxis_Pathway |
| GLUCOSE_UP | GLUCOSE_UP |
| IL_13 | ST_IL_13_PATHWAY |
| Interleukin_13 | ST_Interleukin_13_Pathway |
| Krebs-TCA | Krebs-TCA_Cycle |
| MAP_kk | MAP_kinase_kinase_activity |
| ORTHOS | AR_ORTHOS_MAPPED_TO_U133_VIA_NETAFFX |
| TCA | TCA |
| TESTO_NET | AR_MOUSE_PLUS_TESTO_FROM_NETAFFX |
| bcl2family | bcl2family_and_reg_network |
| cacam | cacamPathway |
| chrebp | chrebpPathway |
| D4gdi | d4gdiPathway |
| fxr | fxrPathway |
| hivnef | hivnefPathway |
| kreb | krebPathway |
| lechtin | lechtinPathway |
| leptin | leptinPathway |
| lymphocyte | lymphocytePathway |
| mitochondria | mitochondriaPathway |
| no1 | no1Pathway |
| plce | plcePathway |
| proteasome | proteasomePathway |
| sodd | soddPathway |
| tnfr1 | tnfr1Pathway |
| ureacycle | ureacyclePathway |
| Aminosugar | MAP00530_Aminosugars_metabolism |
| CYCLIN_EXPR | SA_REG_CASCADE_OF_CYCLIN_EXPR |
| EGFsignal | EGF_receptor_signaling_pathway |
| GPI7TRS | ST_G_Protein_Independent_7_Transmembrane_Receptor_Signaling |
| GPI7T | ST_G_Protein_Independent_7_Transmembrane |
| KRAS | KRAS_TOP100_KNOCKDOWN |
| Purine | MAP00230_Purine_metabolism |
| Vitcb | vitcbPathway |
| Starch | MAP00500_Starch_and_sucrose_metabolism |
| Arap | arapPathway |
| Glycosaminoglycan | MAP00531_Glycosaminoglycan_degradation |
| Erbb4 | erbb4Pathway |
| tgfb | tgfbPathway |
| ptc1 | ptc1Pathway |
| FRASOR_UP | FRASOR_ER_UP |

**Figure 12: PAGE pathway abbreviations.**

| **Pathway abbreviation** | **Pathway name** |
| --- | --- |
| ANDROGEN | ANDROGEN_GENES_FROM_NETAFFX |
| Bile_acid | MAP00120_Bile_acid_biosynthesis |
| Butanoate | MAP00650_Butanoate_metabolism |
| Fattyacidbsy | MAP00062_Fatty_acid_biosynthesis_path_2 |
| Glyoxylate | MAP00630_Glyoxylate_and_dicarboxylate_metabolism |
| KET | KET |
| Propanoate | MAP00640_Propanoate_metabolism |
| Valine | MAP00280_Valine_leucine_and_isoleucine_degradation |
| akap96 | akap96Pathway |
| akapCentrosome | akapCentrosomePathway |
| app | appPathway |
| etc | etcPathway |
| glycolysis | glycolysisPathway |
| mta3 | mta3Pathway |
| par1 | par1Pathway |
| tob1 | tob1Pathway |
| AlaAsp | MAP00252_Alanine_and_aspartate_metabolism |
| FAS | SA_FAS_SIGNALLING |
| G_alpha_5 | ST_G_alpha_5_Pathway |
| G_alpha_i | ST_G_alpha_i_Pathway |
| Glutamate | MAP00251_Glutamate_metabolism |
| Bcl2family | bcl2family_and_reg_network |
| cdk5 | cdk5Pathway |
| cpt | cptPathway |
| CRCELLCYC | CR_CELL_CYCLE |
| mRNA_binding | mRNA_binding_activity |
| muscle_myosin | muscle_myosin |
| FRASOR_DOWN | FRASOR_ER_DOWN |
| FETAL_LIVER | FETAL_LIVER_HS_ENRICHED_TF_JP |
| Akap13 | akap13Pathway |
| Igf1mtor | igf1mtorPathway |
| BRCA_DOWN | BRCA_DOWN |
| CR_TRANS | CR_TRANSCRIPTION_FACTORS |
| CitrateTCA | MAP00020_Citrate_cycle_TCA_cycle |
| HTERT_DOWN | HTERT_DOWN |
| Fructose | MAP00051_Fructose_and_mannose_metabolism |
| G13 | G13_Signaling_Pathway |
| Ga13 | ST_Ga13_Pathway |
| Gaq | ST_Gaq_Pathway |
| Glycans | MAP00512_O_Glycans_biosynthesis |
| Glycine | MAP00260_Glycine_serine_and_threonine_metabolism |
| Keratan | MAP00533_Keratan_sulfate_biosynthesis |
| Krebs-TCA | Krebs-TCA_Cycle |
| N_Glycans | MAP00510_N_Glycans_biosynthesis |
| Pentose | MAP00030_Pentose_phosphate_pathway |
| RAP_DOWN | RAP_DOWN |
| Steroid | MAP00140_C21_Steroid_hormone_metabolism |
| TCA | TCA |
| TRKA | SA_TRKA_RECEPTOR |
| cacam | cacamPathway |
| caspase | caspasePathway |
| sodd | soddPathway |
| cell_adh | cell_adhesion_molecule_activity |
| cell_adhact | cell_adhesion_receptor_activity |
| hivenf | hivnefPathway |
| hsp27 | hsp27Pathway |
| parkin | parkinPathway |
| mitochondria | mitochondriaPathway |
| gcr | gcrPathway |
| eif2 | eif2Pathway |
| il1r | il1rPathway |
| ketonebodies | ketonebodiesPathway |
| malate | malatePathway |
| mtor | mtorPathway |
| nkt | nktPathway |
| pparg | ppargPathway |
| slrp | slrpPathway |
| INSULIN_2F_UP | INSULIN_2F_UP |
| TRANSPORT | CR_TRANSPORT |
| arenrf2 | arenrf2Pathway |
| stress | stressPathway |
| tall1 | tall1Pathway |
| vitcb | vitcbPathway |
| Terc | tercPathway |
| cbl | cblPathway |

**Figure 13: PAGE pathway abbreviations.**

| **Pathway abbreviation** | **Pathway name** |
| --- | --- |
| Complement | Complement_Activation_Classical |
| cd40 | cd40Pathway |
| ets | etsPathway |
| gs | gsPathway |
| IL-17 | IL-17Pathway |
| nfkb | nfkbPathway |
| pkc | pkcPathway |
| Shh | Shh_lisa |
| ldl | ldlPathway |
| electron transporter | electron transporter activity |
| ANDROGEN | ANDROGEN_GENES_FROM_NETAFFX |
| Bile_acid | MAP00120_Bile_acid_biosynthesis |
| Butanoate | MAP00650_Butanoate_metabolism |
| FAS | SA_FAS_SIGNALLING |
| cpt | cptPathway |
| ROS | ROS |
| Wnt_Ca2 | ST_Wnt_Ca2_cyclic_GMP_Pathway |
| hbx | hbxPathway |
| intrinsic | intrinsicPathway |
| cysteine | MAP00272_Cysteine_metabolism |
| chrebp | chrebpPathway |
| erbb4 | Erbb4Pathway |
| lechtin | lechtinPathway |
| glucose_up | GLUCOSE_UP |
| FA biosynth | MAP00062_Fatty_acid_biosynthesis_path_2 |
| Glyoxylate | MAP00630_Glyoxylate_and_dicarboxylate_metabolism |
| KET | KET |
| Propanoate | MAP00640_Propanoate_metabolism |
| Valine metab | MAP00280_Valine_leucine_and_isoleucine_degradation |
| etc | etcPathway |
| glycolysis | glycolysisPathway |
| tob1 | tob1Pathway |
| FAS | SA_FAS_SIGNALLING |
| G_alpha_i | ST_G_alpha_i_Pathway |
| cpt | cptPathway |
| muscle_myosin | muscle_myosin |
| FRASOR | FRASOR_ER_DOWN |
| FETAL_LIVER | FETAL_LIVER_HS_ENRICHED_TF_JP |
| Igf1mtor | igf1mtorPathway |
| CR_TRANS | CR_TRANSCRIPTION_FACTORS |
| CitrateTCA | MAP00020_Citrate_cycle_TCA_cycle |
| G13 | G13_Signaling_Pathway |
| O_Glycans | MAP00512_O_Glycans_biosynthesis |
| Krebs-TCA | Krebs-TCA_Cycle |
| Steroid | MAP00140_C21_Steroid_hormone_metabolism |
| TCA | TCA |
| TrkA | SA_TRKA_RECEPTOR |
| cacam | cacamPathway |
| sodd | soddPathway |
| cell_adhmol | cell_adhesion_molecule_activity |
| cell_adhrec | cell_adhesion_receptor_activity |
| hivenf | hivnefPathway |
| hsp27 | hsp27Pathway |
| mitochondria | mitochondriaPathway |
| gcr | gcrPathway |
| eif2 | eif2Pathway |
| ketone | ketonebodiesPathway |
| malate | malatePathway |
| pparγ | ppargPathway |
| slrp | slrpPathway |
| stress | stressPathway |
| vitcb | vitcbPathway |

**Figure 14: PAGE pathway abbreviations.**

| **Pathway abbreviation** | **Pathway name** |
| --- | --- |
| starch | MAP00500_Starch_and_sucrose_metabolism |
| ps1 | ps1Pathway |
| atm | atmPathway |
| DAG1 | SA_DAG1 |
| glutathione | MAP00480_Glutathione_metabolism |
| erbb4 | erbb4Pathway |
| IL-17 | IL-17Pathway |
| gata3 | gata3Pathway |
| csk | cskPathway |
| ami | amiPathway |
| BCR_SIG | SIG_BCR_Signaling_Pathway |
| hbx | hbxPathway |
| methane | MAP00680_Methane_metabolism |
| inflam | inflamPathway |
| MMP_CYTOKINE | SA_MMP_CYTOKINE_CONNECTION |
| CO2-fixation | MAP00720_Reductive_carboxylate_cycle_CO2_fixation |
| GPI7T | ST_G_Protein_Independent_7_Transmembrane |
| GPI7TRS | ST_G_Protein_Independent_7_Transmembrane_Receptor_Signaling |
| CYCLIN | SA_REG_CASCADE_OF_CYCLIN_EXPR |
| SIG_CHEMOTAXIS | SIG_CHEMOTAXIS |
| mta3 | mta3Pathway |
| app | appPathway |
| eif2 | eif2Pathway |
| slrp | slrpPathway |
| ketone bodies | ketonebodiesPathway |
| malate | malatePathway |
| glycolysis | glycolysisPathway |
| Bile acid | MAP00120_Bile_acid_biosynthesis |
| IGF-1-mTOR | Igf1mtorPathway |
| Ala-Asp | MAP00252_Alanine_and_aspartate_metabolism |
| Bcl2-family | bcl2family_and_reg_network |
| steroid | MAP00140_C21_Steroid_hormone_metabolism |
| FAS | SA_FAS_SIGNALLING |
| cpt | cptPathway |
| Dictyostelium-cAMP  chemotaxis | Dictyostelium_discoideum_cAMP_Chemotaxis_Pathway |
| AKAP-13 | Akap13Pathway |
| O_glycans | MAP00512_O_Glycans_biosynthesis |
| cacam | cacam |
| ANDROGEN | ANDROGEN_GENES_FROM_NETAFFX |
| FETAL_LIVER | FETAL_LIVER_HS_ENRICHED_TF_JP |
| etc | etcPathway |
| CR_TRANSPORT | CR_TRANSPORT |
